# T Lymphocytes Influence the Mineralization Process of Bone

**DOI:** 10.3389/fimmu.2017.00562

**Published:** 2017-05-24

**Authors:** Thaqif El Khassawna, Alessandro Serra, Christian H. Bucher, Ansgar Petersen, Claudia Schlundt, Ireen Könnecke, Deeksha Malhan, Sebastian Wendler, Hanna Schell, Hans-Dieter Volk, Katharina Schmidt-Bleek, Georg N. Duda

**Affiliations:** ^1^Experimental Trauma Surgery, Faculty of Medicine, Justus-Liebig University, Giessen, Germany; ^2^German Arthritis Research Center (DRFZ), Berlin, Germany; ^3^Julius Wolff Institute, Center for Musculoskeletal Surgery, Charité – Universitätsmedizin Berlin, Berlin, Germany; ^4^Berlin-Brandenburg Center for Regenerative Therapies, Charité – Universitätsmedizin Berlin, Berlin, Germany; ^5^Institute of Medical Immunology, Charité – Universitätsmedizin Berlin, Berlin, Germany

**Keywords:** bone healing, collagen I, T lymphocytes, immune cells, mineralization

## Abstract

Bone is a unique organ able to regenerate itself after injuries. This regeneration requires the local interplay between different biological systems such as inflammation and matrix formation. Structural reconstitution is initiated by an inflammatory response orchestrated by the host immune system. However, the individual role of T cells and B cells in regeneration and their relationship to bone tissue reconstitution remain unknown. Comparing bone and fracture healing in animals with and without mature T and B cells revealed the essential role of these immune cells in determining the tissue mineralization and thus the bone quality. Bone without mature T and B cells is stiffer when compared to wild-type bone thus lacking the elasticity that helps to absorb forces, thus preventing fractures. In-depth analysis showed dysregulations in collagen deposition and osteoblast distribution upon lack of mature T and B cells. These changes in matrix deposition have been correlated with T cells rather than B cells within this study. This work presents, for the first time, a direct link between immune cells and matrix formation during bone healing after fracture. It illustrates specifically the role of T cells in the collagen organization process and the lack thereof in the absence of T cells.

## Introduction

Bone is capable of regeneration, but the process that leads to scarless restoration of form and function is highly complex and prone to failure (1). Even today delayed and non-union following bone injury represent a clinical problem that results in strongly reduced quality of life for affected patients (2). Despite the progress in characterizing molecular and cellular elements of the bone regeneration cascade, the distinct aspects that are altered in compromised patients remain so far unclear. The need to understand the correlation of systemic diseases that influence the immune reaction to bone healing remains.

Only recently the tight interplay of the skeletal and immune system has been described and recognized as a key element of the bone healing cascade (3). The current understanding of this interplay, however, is rather controversially discussed. On one hand, lymphopenic mice display a seemingly better healing after injury (4). On the other hand, several studies attribute either a positive or negative role to T and B cells in bone biology, autoimmunity, and fracture repair. Activated T cells are responsible for causing bone loss during rheumatoid arthritis (5) and postmenopausal osteoporosis (6), and CD8^+^ memory/effector T cells have been linked to delayed bone healing (7).

It is also known that fracture repair starts with a pro-inflammatory reaction which is essential for triggering the healing cascade (8), that macrophages are essential for successful bone regeneration (9), that osteoblast maturation is triggered by T-lymphocytes (10), and that regulatory T cells cause higher bone mass (11) and enhance bone repair (12). B and T cells are producers of osteoprotegerin (OPG) and receptor activator of NF-kB ligand (RANKL) influencing the osteoclastogenesis, and they are present in high numbers during the remodeling process that reorganizes bone structure toward its final mechanical strength and competence (13). The above-described detrimental or beneficial effects address different phases of bone formation and healing, but their detailed interplay remains so far unknown. Specifically, the distinct role of T and B cells during bone healing appears to be unclear. From previous experiments, we could identify that the absence of mature B and T lymphocytes accelerated fracture healing (4).

In this work, we ascertain whether such changes in bone repair are dependent on either T or B cells. Furthermore, we analyzed the implications of a changed matrix formation on the quality of newly generated bone and on the underlying osteoblast activity.

## Materials and Methods

### Experimental Design

To assess the global role of T and B cells in bone function, structure, and regeneration process, a mouse model of recombination activating gene 1 homozygous knockout (RAG1^−/−^) (C57BL/6N background; Bundesinstitut für Risikobewertung, Berlin, Germany) was used. RAG1^−/−^ mice lack both mature T and B cells (14). The differentiation of T and B cells in these mice is arrested at an early stage due to their inability to perform V(D)J recombination. Discrepancies in bone architecture and function between the RAG1^−/−^ mice and wild-type (WT) controls (C57BL/6N, Charles River Laboratories, Wilmington, MA, USA) were assessed on various levels: bone biomechanics, by biomechanical torsional testing, morphology by micro computed tomography (microCT) analysis and histological staining, and differential gene expression by microarray, qPCR analysis, and flow cytometry.

Bone healing phases in both groups were investigated by creating a unilateral closed fracture in the diaphysis of the left femur according to Bonnarens and Einhorn (15). Fracture healing was assessed on an 8-week-old male at (day = D) D3, D7, D14, D21, and D28 after surgery.

Furthermore, the individual role of each T and B cells in the cartilaginous callus formation (D7) was investigated. A group of T cell receptor β and δ chain homozygous knockout (TCRβδ^−/−^) mice (16) (*n* = 8) (AG Löhning, Charité – Universitätsmedizin Berlin) was investigated at D7 post fracture. Through knockout of genes in the beta- and delta-chain of the T cell receptor, T cells in these animals were unable to express functional receptors that induce cell apoptosis. Bone healing without the influence of T cell was investigated in this group. On the other hand, a second group of immunoglobulin heavy chain, joining region homozygous knockout (JHT^−/−^) mice (17) (*n* = 8) (Bundesinstitut für Risikobewertung, Berlin, Germany) was investigated 7 days post fracture to analyze healing without B cells. These mice harbor a deletion mediated by cre-loxP resulting in the loss of the exons encoding the joining region and the intron enhancer of the immunoglobulin heavy chain locus (IgH). The JHT^−/−^ mice fail to produce functional B-cells as they lack the gene for the assembly of the heavy chain for the production of antibodies.

Adhering to the 3R principles, sample sizes were kept as small as possible—results were confirmed by using different analyzing methods.

### Surgical Procedure

During the operation, mice were anesthetized with 2.5% isoflurane/oxygen and received pain medication [buprenorphine 1 mg/kg BW intraperitoneally (i.p.), Reckitt Benckiser, Mannheim, Germany]. After opening the skin and the knee joint the patella was dislocated to the lateral side. Through the distal end of the femur a passage for the intramedullary pin was created using a hollow needle (Microlance 3, 0.55 mm × 25 mm, BD Drogheda, Ireland). This needle was removed and an intramedullary pin (Thermo spinal needle 17, 0.5 mm × 0.9 mm, TERUMO EUROPE N. V., Leuven, Belgium) was inserted into the bone marrow cavity.

All experiments were carried out with ethical permission according to the policies and principles established by the Animal Welfare Act, the National Institutes of Health Guide for Care and Use of Laboratory Animals, and the National Animal Welfare Guidelines and were approved by the local legal representative animal rights protection authorities (Landesamt für Gesundheit und Soziales Berlin: G 0206/08).

### Biomechanical Testing

To study the initial mechanical properties, torsional testing was carried out on the intact contralateral femora of WT and RAG1^−/−^ animals (*n* = 18 per group). The proximal and distal ends of the femur were embedded into the holding pots using polymethyl methacrylate. Using a laser line, femora were aligned parallel to the loading axis and centered into the holding pots. Bones were brought to the point of failure under torsional loading setup of 0.5°/s and 0.3 N axial preload (BOSE ElectroForce 3200 test system, Eden Prairie, MN, USA). Torque of failure (M_max), degree of torsion (angle-at-max), energy at break, and stiffness were evaluated for the intact contralateral bones.

### X-Ray Micro Computed Tomography (microCT) Analyses

Mineralized tissue has been analyzed using microCT. Therefore, the femur was isolated from untreated animals or from the contralateral side of animals that received a fracture (WT *n* = 56, RAG1^−/−^ *n* = 39). Femora were scanned with a fixed isotropic voxel size of 10.5 µm (VivaCT, Scanco Medical AG^®^, Switzerland, 70 kVp, 114 µA). The scan axis equaled the diaphyseal axis of the femora. A diaphyseal volume of interest with a size of 630 slices was analyzed with a global threshold of 190 mg HA/cm^3^. All analyses were performed on the digitally extracted callus tissue using 3D distance techniques (Scanco^®^ software, Switzerland) (18).

### Sample Preparation for Histological Analyses

To analyze tissue formation over the course of healing a histological analysis was performed according to the targeted test. Femora were harvested at D3, D7, D14, D21, and D28 of healing from the WT and RAG1^−/−^ mice for paraffin embedding (*n* = 8 per time point and group). Briefly, bones were harvested with little surrounding muscle tissue and fixated in 4% paraformaldehyde (PFA) at 4°C for 48 h. Afterward, decalcification was done for 3 weeks in a 1:1 solution of 4% PFA and 14% EDTA at 4°C. After dehydration bones were embedded in paraffin and cut into 4 µm sagittal slices.

Furthermore, femora of WT and RAG1^−/−^ mice were also harvested at 7, 14, and 21 days post fracture (*n* = 3 per group and time point) for plastic embedding. Briefly, femora were fixed with 4% PFA for 48 h, dehydrated, and plastic embedded (Technovit^®^ 9100 neu, Heraeus Kulzer GmbH, Wehrheim, Germany), and then 7 µm sagittal slices were prepared.

Another set of animals from WT, JHT^−/−^, and TCRβδ^−/−^ groups (*n* = 3 per group) were collected at 7 days post fracture processed for cryo-sectioning. Briefly, bones were harvested and fixed in 4% PFA for 2 h at 4°C. Afterward, the bones were treated in increasing concentrations of sucrose solution 10%, 20%, and finally 30% for 24 h each at 4°C. Bones were then embedded in SCEM cryo-embedding medium (Section Lab Co Ltd., Yokohama, Japan). Cryostat sections of 7 µm were cut sagittal using the Leica CM3050S Cryotom (Leica Microsystems, Nussloch GmbH, Germany) and Cryofilm [Cryofilm type II C (9), Section Lab Co Ltd., Yokohama, Japan].

### Histological Assessment and Histomorphometry

Paraffin- and cryo-sections were used for a general overview of the different tissues that are involved in fracture healing using Movat’s pentachrome staining. The Movat’s pentachrome is a combination of alcian blue solution, Weigert’s iron hematoxylin, brilliant crocein acid fuchsin, phosphotungstic acid, and safran du Gatinais solution. The stain differentiates cartilage formation and hypertrophy (green), matrix mineralization (yellow), connective tissue (light blue), muscle (orange), bone marrow (purple), and was used for histomorphometric analyses (19), using a computerized histomorphometric analysis with an image analysis system (KS400 3.0, Zeiss, Eching, Germany).

### Triple Fluorochrome Labeling

To label the mineral formation near the fracture gap on the endosteal and periosteal regions *in vivo* fluorescence dyes were injected i.p., (*n* = 3). Dyes were dissolved in 1.4% NaHCO_3_ and injected with respect to the time point of euthanasia: calcein blue (blue fluorescence, 30 mg/kg BW, M1255, Sigma, Hamburg, Germany) 12 days pre-euthanasia, calcein green (20 mg/kg i.p.; C0875, Sigma, Hamburg, Germany) 7 days pre-euthanasia, and Alizarin red (30 mg/kg i.p.; A3882, Sigma, Hamburg, Germany) 2 days pre-euthanasia. After sacrifice, femora were dissected and embedded in PMMA as described above. 7 µm thick sections were inspected using fluorescent microscopy and analyzed semiautomated using the provided software (Axioskop 40, and AxioVision software; Carl Zeiss MicroImaging GmbH, Göttingen, Germany).

### Immunohistochemical (IHC) and Fluorescent IHC Staining of Bone Matrix and Cells

Immunohistochemical staining was performed on paraffin sections to visualize collagen type I (ColI) at 7 days post fracture (*n* = 3) in WT and RAG1^−/−^ animals. Briefly, deparaffinized sections were incubated with hyaluronidase for antigen retrieval, rinsed in phosphate-buffered saline (PBS) and incubated with the primary antibody (anti-type I collagen rabbit, C020121, Biologon, 1:500 dilution) overnight at 4°C. Using the avidin/biotin complex method (Alkaline Phosphatase Universal, AK-5200, Vectastain ABC Kit, Vector, CA, USA) binding of the secondary antibody (biotinylated anti-rabbit IgG, made in goat, BA-1000, Vector laboratories, CA, USA) to the primary was visualized by AP substrate (Alkaline Phosphatase Substrate Kit 1, SK-5100, Vector). Counterstaining was performed with hematoxylin. As a negative control, sections were processed for each sample in the absence of the suitable primary antibody.

Fluorescent IHC was performed on cryo-sections of JHT^−/−^, TCRβδ^−/−^, and WT at D7 post fracture (*n* = 3, for each group). The approach was used to visualize the following components: 1) B cells using anti-mouse B220 (B220/CD45R Alexa Fluor 488-conjugated antibody, Clone # RA3-6B2, R&D Systems GmbH Wiesbaden, Germany); 2) T cells using anti-mouse CD3e (CD3e PE, Clone # eBio500A2, eBioscience, CA, USA); 3) osteocalcin (Ocn) using anti-mouse (rabbit polyclonal, Enzo Life Sciences, cat. no ALX-210-333, 1:4,000); and 4) ColI using anti-mouse ColI (rabbit polyclonal, Bio-Rad). Briefly, sections were brought to room temperature (RT) and washed with PBS. An initial blocking step was performed by applying a solution of PBS containing 5% FCS, 0.1% TWEEN-20, and 10% rat serum for 30 min. For Ocn and ColI staining, sections were blocked with un-labeled Donkey gamma globulin (Jackson). All subsequent washing steps were done using PBS with 5% FCS and 0.1% TWEEN-20. All primary antibodies were incubated for 1 h at RT. Secondary antibodies were used (Alexa Fluor 488, A21206, Invitrogen, 1:500; Alexa 647, Invitrogen, 1:600) for detection. DAPI was used to stain nuclei. After staining was completed the slices were mounted with DAKO fluorescent mounting medium (S3023, DAKO, Hamburg, Germany). To analyze the staining a LSM 710 confocal microscope (Carl Zeiss, Jena, Germany) was used, and pictures were taken using the software Zen 2011 (Carl Zeiss MicroImaging GmbH, Göttingen, Germany). Image analysis was performed using the ImageJ software.

Tartrate-resistant acid phosphatase (TRAP) staining was performed to determine osteoclasts. Counterstaining was done with methyl green. TRAP-positive cells with >3 nuclei and positioned on the bone surface were considered osteoclasts and included in the evaluation. A total of *N* = 8 animals were analyzed per group, osteoclast numbers were normalized against the bone surface in the analyzed region of interest.

### Second Harmonic Generation (SHG) Microscopy

Plastic-embedded samples (WT = 3, RAG1^−/−^ = 4, D7) were used to visualize collagen fibrils in WT and RAG1^−/−^ mice *via* SHG imaging. Collagen fibrils exhibit endogenous SHG signals arising from their well-known non-centrosymmetric molecular structure (20, 21). Imaging was performed using a Leica SP5 II microscope (Leica Microsystems, Wetzlar, Germany). The SHG signal was generated using a Mai Tai^®^ HP Ti:Sapphire oscillator (Spectra Physics, Stahnsdorf, Germany) with 100 fs pulse width at 80 MHz and wavelength of 910 nm. The SHG collagen signal was detected in the range of 450–460 nm. Z-Stacks were recorded with 4 µm *z*-spacing using 25× water immersion objectives with numerical apertures of 0.95. Overview images were created *via* image stitching and maximal intensity projections of z-stacks.

### Microarray Analysis

To support our macroscopic findings a gene expression analysis was performed.

#### mRNA Isolation and Expression Analysis

For RNA preparation from fracture callus, the soft tissue was removed and the fractured bone samples including 1 mm diaphyseal bone on either side of the fracture (*n* = 5 per group and time point) were taken. Same region was also collected from intact femora from both groups to assess indigenous differences. Samples were immediately snap frozen in liquid nitrogen and stored at −80°C until further use. Bones were pulverized in liquid nitrogen and transferred to TRIzol (Invitrogen Life Technologies, Germany) and RNA was isolated according to the manufacturer’s protocol with DNAse I (Invitrogen Life Technologies) digestion included. A total of 150 ng RNA per sample was pooled in a given group (*n* = 5 per group). The pooled RNA mixture was distributed as 200 ng samples per tube for technical triplicate in the subsequent microarray analysis.

#### Microarray Hybridization

Whole genome expression profiling was performed using Illumina’s MouseRef-8 v2.0 Expression BeadChips (Illumina, Ambion, TX, USA). Processing RNA for hybridization, including cRNA synthesis and labeling, was done according to standard protocols described in the instruction manual of Illumina TotalPrep RNA amplification kit (Illumina, Ambion, TX, USA). Hybridization of the labeled and fragmented cRNA to the microarray and subsequent staining, washing, and scanning of the arrays was done according to the Illumina Whole-Genome Gene Expression direct Hybridization Assay guide.

#### Microarray Evaluation

The arrays were pre-processed and normalized using R (22), the bead array (23), and Bioconductor (24) packages. Between arrays, quintile was normalized using the Lumi package (25). Quality assessment of the microarray data was performed by computing the mean Pearson correlation between each array and every other array in the test database. Exclusion of arrays was unnecessary as they did not exceed the mean correlation cutoff of 0.6. Pearson correlation of each pair was used to evaluate replicates. Mean correlation cutoff was set to 0.9—no replicate removal was necessary. Differences between gene expression at each time point in WT and RAG1^−/−^ was calculated using a two-tailed *t*-test. The *p*-values were alpha-error adjusted using the Benjamini–Hochberg method. Fold changes (FCs) were calculated from comparison of the mean expressions between the control (WT) and the treatment (RAG1^−/−^) group. Differentially expressed genes were visualized in a heatmap after applying a *t*-test analysis to reveal the significant gene regulations for each analyzed time point. Significance FC cutoff of >1 and Benjamini and Hochberg-corrected *p*-values (*p*.BH) <0.05 was followed for the guilt-by-association (GBA) analysis and a cutoff of FC > 1 and *p*.BH < 0.01 for the panel analysis. Genes were then clustered (arranged) according to their expression pattern over the course of healing. Gene lists of the expression clusters were then compared to literature and NCBI-DAVID (an online server for the gene annotation) to characterize the major biological processes, molecular functions, and cellular components for these genes.

### Quantitative Reverse Transcription Polymerase Chain Reaction (qRT-PCR)

To evaluate the differential expression of collagen I and bone markers [OPG, RANKL, Runt-related transcription factor 2 (RUNX2)] a qRT-PCR was performed. Femora from WT, RAG1^−/−^, TCRβδ^−/−^, and JHT^−/−^ (*n* = 4) were harvested and instantly frozen in liquid nitrogen and then stored at −80°C until processing. RNA was extracted using a Trizol/chloroform (TRIzol^®^ Reagent, Invitrogen, 15596-018; chloroform for molecular biology, VWR, SIALC2432-25ML) and the RNAeasy kit [RNeasy Mini Kit (74104), RNase Free DNase Set (79254), Nuclease Free Water (129114), QIAGEN]. A total of 100 ng RNA per sample have been reverse transcribed using random primer [Random Primers, Invitrogen, 48190-011; Set of four dNTPs (100 mM), BIORON, 110011; Recombinant RNasin^®^ Ribonuclease Inhibitor inkl. M-MLV RT 5× Reactionbuffer, Promega, N2511; M-MLV Reverse Transcriptase RNase H Minus, Point Mutant, Promega, M3683; Ribonuclease H, Promega, M4285]. Primers were tested for the annealing temperature and efficiency prior to use: mCOL1a1 ase: 5′-gttccaggcaatccacgag-3′, mCOL1a1 se: 5′-ggtccacaaggtttccaagg-3′; mOPG ase: 5′-ctgctctgtggtgaggttcg-3′, mOPG se: 5′-agctgctgaagctgtggaaa-3′; mRANKL ase: 5′-cgaaagcaaatgttggcgta-3′, mRANKL se: 5′-gcacacctcaccatcaatgc-3′; mRUNX2 ase: 5′-tgtctgtgccttcttggttcc-3′, and mRUNX2 se: 5′-cgaaatgcctccgctgttat-3′. Cyclophilin A was used as a house keeping gene. PCR cycle: 40 cycles each consisting of 95°C, 30 s, 62°C, 30 s, 72°C, 30 s. Evaluation was done using the 2^−ΔCt^ method.

### Flow Cytometry

Splenocytes were harvested from WT, RAG1^−/−^, TCRβδ^−/−^, and JHT^−/−^ (*n* = 4) and brought into single cell suspension. Cells were washed and resuspended in FACS buffer (PBS/0.5% BSA/0.1% sodium acid). Staining with the fluorescent coupled antibodies was performed for live/dead (Invitrogen LIVE/DEAD fixable blue dead cell stain kit, for UV excitation), CD45 (30-F11, V500, BD Horizon), CD3e (145-2C11, PerCP/Cy5.5, BioLegend), and CD8a (53-6.7, BV785, BioLegend). The FACS analysis was done with a BD LSRFortessa (BD Deutschland GmbH). The FlowJo software (TreeStar Inc., Ashland, OR, USA) was used for data evaluation. Gating strategy: lymphocytes, single cells, live cells, CD45-positive cells, CD3e-positive cells, CD8a-positive cells to determine CD8^+^ T cell percentage in the respective mouse strains.

### Protein Expression Analysis

Conditioned media of LPS-activated splenocytes of WT, TCRβδ^−/−^, JHT^−/−^, and RAG1^−/−^ mice (*n* = 4) were pooled and analyzed toward their TNFα and interleukin 10 (IL-10) concentrations. Mouse TNFα ELISA Ready-SET-Go!^®^ and Mouse IL-10 ELISA Ready-SET-Go!^®^ kits (eBioscience, San Diego, CA, USA) were carried out after manufacturer’s recommendations. Samples were incubated overnight at 4°C, final staining reactions were stopped with 1 M H_3_PO_4_ and absorbance was read at 450 nm with 570 nm reference wavelength with Tecan Infinite M200 PRO (Tecan, Männedorf, Switzerland) and analyzed with i-control 1.9 software (Tecan).

### Cell Culture

Isolated murine MSCs were cultured in chamber slides with expansion medium for 1 day. Then osteogenic differentiation medium was added which included conditioned medium (1:3 dilution) from LPS (1 µg/ml)-activated splenocytes of WT, RAG1^−/−^, TCRβδ^−/−^, and JHT^−/−^ mice (*n* = 4). Medium change was done twice per week for 14 days. Collagen fibers were stained with Sirius red and analyzed under polarized light.

### Statistical Analysis

Tests for statistical significance were performed in SPSS 21.0 (IBM, CA, USA). Data from histomorphometry, biomechanics, and microCT analyses were explored for normality by examining skewness and kurtosis. Data were not normally distributed. Therefore, Bonferroni *post hoc* corrected Mann–Whitney *U* test was used to test the significance between the two groups in a given time point. *p*-Values of <0.05 were chosen to indicate an exact two-tailed significance. Data were presented as box plots (median and interquartile range) or bar graphs (mean ± SEM).

In the analyses of statistical significance, *n* ≥ 5 animals were included per group. This was the lowest number of samples needed for the present study based on *a priori* assumption for qualitative statements. For descriptive analyses, samples sizes of *n* < 5 were used. No experimental animals were excluded from the analysis.

## Results

RAG1^−/−^ mice are in their gross appearance similar to WT. Without mature T and B cells bone developmental processes, however, are altered and this alteration in organogenesis is reflected in fracture healing. Detailed analysis of structural parameters, functional competence, and expression profiling in intact RAG1^−/−^ bone was performed (Figure S1 in Supplementary Material).

### Morphological Differences between RAG1^−/−^ and WT Bones

**Figure 1:**
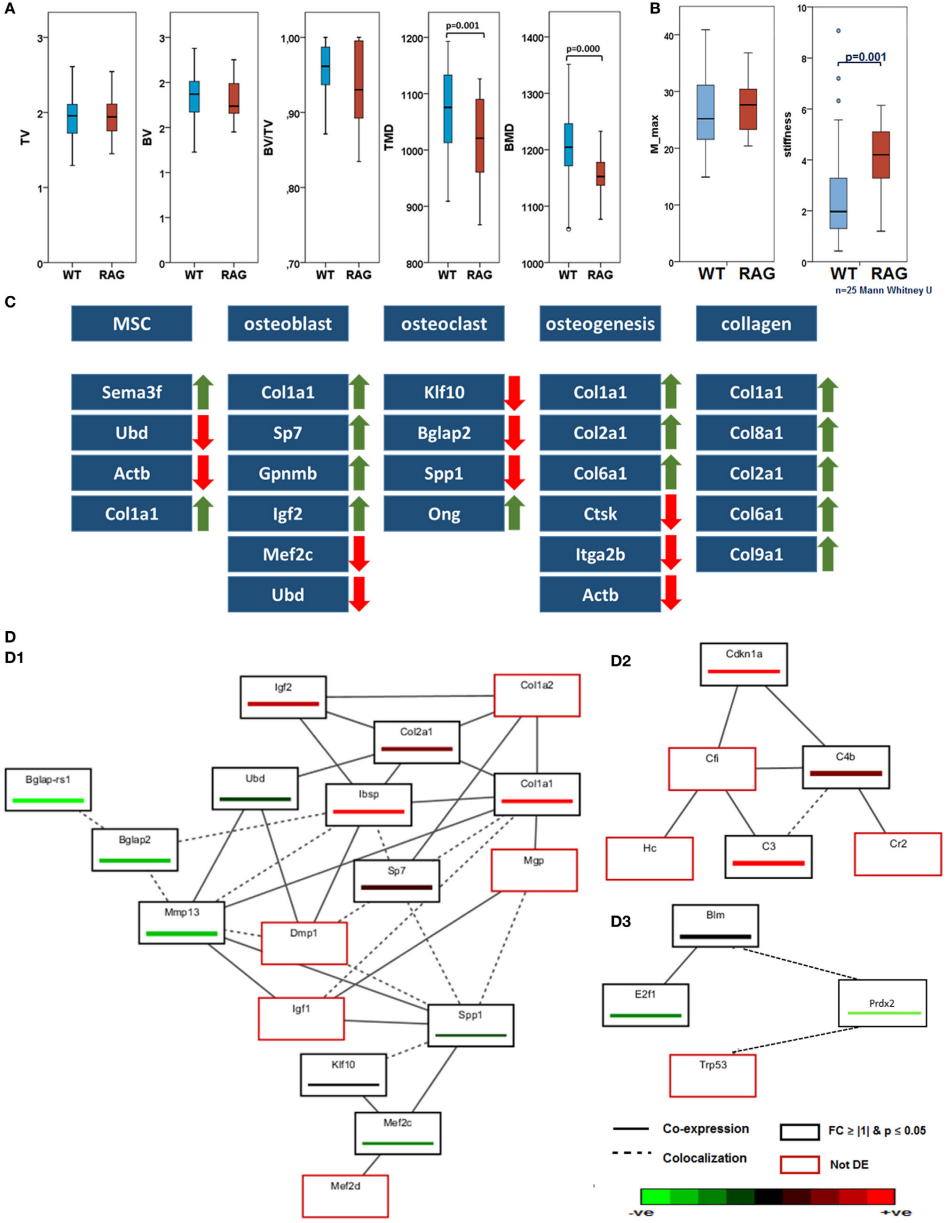
**Intact bone of wild-type (WT) and recombination activating gene 1 homozygous knockout (RAG1^−/−^) while having the same morphology difference in their biomechanical competence and gene expression: (A) micro computed tomography analyses showed no differences in total volume (TV), bone volume (BV), and BV/TV**. In those parameters describing the bone quality, tissue mineral density (TMD, *p* = 0.001) and bone mineral density (*p* = 0.000), however, significant differences were found between WT and RAG^−/−^ animals with RAG^−/−^ showing a decreased TMD and BMD (WT *n* = 56, RAG^−/−^*n* = 39). **(B)** The torque of failure (M_max) is the same in untreated bone of WT and RAG1^−/−^ animals. However, the RAG1^−/−^ bones show significantly higher stiffness when compared with WT bones. **(C)** Differentially expressed genes correlated with T and B cells were analyzed in intact bones. Expression changes were seen on cellular and extracellular matrix (ECM) levels and in bone homeostasis and osteogenic processes. Major expression changes were seen as an increase in collagen genes expression and their subunits. **(D)** Guilt-by-association analysis correlates genes regulation depending on their coexpression and colocalization. (D.1) differentially expressed genes as described in **(C)** were associated with ECM proteins, (D.2) shows upregulation in B cell-related genes and (D.3) shows downregulation of T cell-regulated genes.

To characterize phenotypic differences between the RAG1^−/−^ and WT, intact bones were investigated using histological and radiological tests. Movat’s pentachrome staining indicated no morphological differences between the femora of both groups. Histomorphological analysis was also performed. However, no significant differences in trabecular number or thickness were found (data not shown). microCT data of unfractured and contralateral bones of WT and RAG1^−/−^ animals showed no differences in total volume, bone volume (BV), or the ratio thereof; however, qualitative bone markers such as tissue mineral density and bone mineral density (BMD) were significantly different (Figure 1A).

### Discrepant Functional Competence in the RAG1^−/−^ Intact Femora

To determine indigenous discrepancies in material properties resulting from the lack of mature T and B cells, intact bones from RAG1^−/−^ and WT animals were subjected to biomechanical torsional testing. While the moment to failure (M_max) did not differ between RAG1^−/−^ and WT (Figure 1B), it became apparent that WT bones endured significantly higher deformation equaled by the angle at maximum force at failure (angle-at-max). This reflects on the energy required to cause the material failure in the form of fracture (energy-to-break), which was lower in RAG1^−/−^ compared to the WT. Therefore, the significantly higher stiffness found in the RAG1^−/−^ compared to the WT controls indicates a stiffer but more brittle bone in RAG1^−/−^.

### Gene Expression Data Reveal Discrepancies in Bone Structure due to the Lack of Mature T and B Cells

Differentially expressed genes associated with a role in bone structure and formation and a correlation with T and B cells were analyzed in intact bones. (Detailed data of the initial discrepancies between both the groups are shown in Table S1 in Supplementary Material.) Microarray data of intact bone between WT and RAG1^−/−^ showed changes in bone homeostasis and osteogenic processes (Figure 1C). Mesenchymal stem cells (MSCs)-related genes were upregulated: the Sema3f gene [a known chemorepulsant from the Semaphorin protein family (26)] and type 1 collagen alpha one (Col1a1). On the other hand, ubiquitin-D [Ubd, a known cell survival factor (27)] and actin beta (Actb, which is involved in MSC differentiation into osteoblasts) were downregulated. The lack of mature T and B cells also affected osteoblasts through four upregulated genes [Col1a1, Sp7 transcription factor (Sp7), Gpnmb also known as osteoactivin, and insulin growth factor 2 (Igf2)] and another two downregulated genes [transcription factor myocyte enhancer factor 2C (Mef2c) and Ubd]. Osteoclast-related genes were mostly downregulated: Kruppel-like factor 10, bone gamma carboxyglutamate protein (Bglap), and osteopontin (Spp1). One gene was upregulated: osteoglycin. This differential expression was reflected in osteogenic processes that showed a downregulation in osteoclast-mediated resorption genes such as cathepsin K, integrin alpha-IIb (also known as CD41) and Actb. However, collagen types correlated with bone matrix formation were upregulated (Col1a1, Col2a1, and Col6a1). This increase in collagen genes expression was also found in other collagen subunits such as Col8a1, Col9a1, and Col10a1.

Guilt-by-association analysis correlates genes regulated in T and B cells and osteogenesis depending on their coexpression and colocalization (Figure 1D). The osteogenic process in the RAG1^−/−^ was compared to the WT. Differentially expressed genes as described in Figure 1C were associated with the following genes: matrix Gla protein, dentin matrix acidic phosphoprotein 1, insulin growth factor 1 (Igf1), myocyte enhancer factor 2D (Mef2d), and Col1a2 (Figure 1, D.1).

Microarray analysis also showed upregulated B cell-related genes (Figure 1, D.2), such as cyclin-dependent kinase inhibitor 1A, complement component 4B, and complement component 3. GBA analysis revealed three non-differentially regulated genes: complement component factor i, complement receptor 2, and hemolytic complement genes were coexpressed with the B cell-related network genes.

Interestingly, T cell-related genes (Figure 1, D.3) were downregulated (Bloom syndrome, E2F transcription factor 1 and peroxiredoxin 2) in the RAG1^−/−^ compared to the WT. Another gene was found to be colocalized with these genes: transformation-related protein 53.

### Bone Healing with and without Mature T and B Cells

To confirm the effects of T and B cells in bone formation processes, bone healing was analyzed in WT and compared to bone healing in RAG1^−/−^ mice: Movat’s pentachrome staining was used to quantify dimensions and distribution of callus tissue during the time course of healing. Histomorphometric methods were used to quantify cartilaginous fractions including zones of hypertrophic chondrocytes as well as ossified regions at the periosteal bone surface area in the fracture callus.

**Figure 2:**
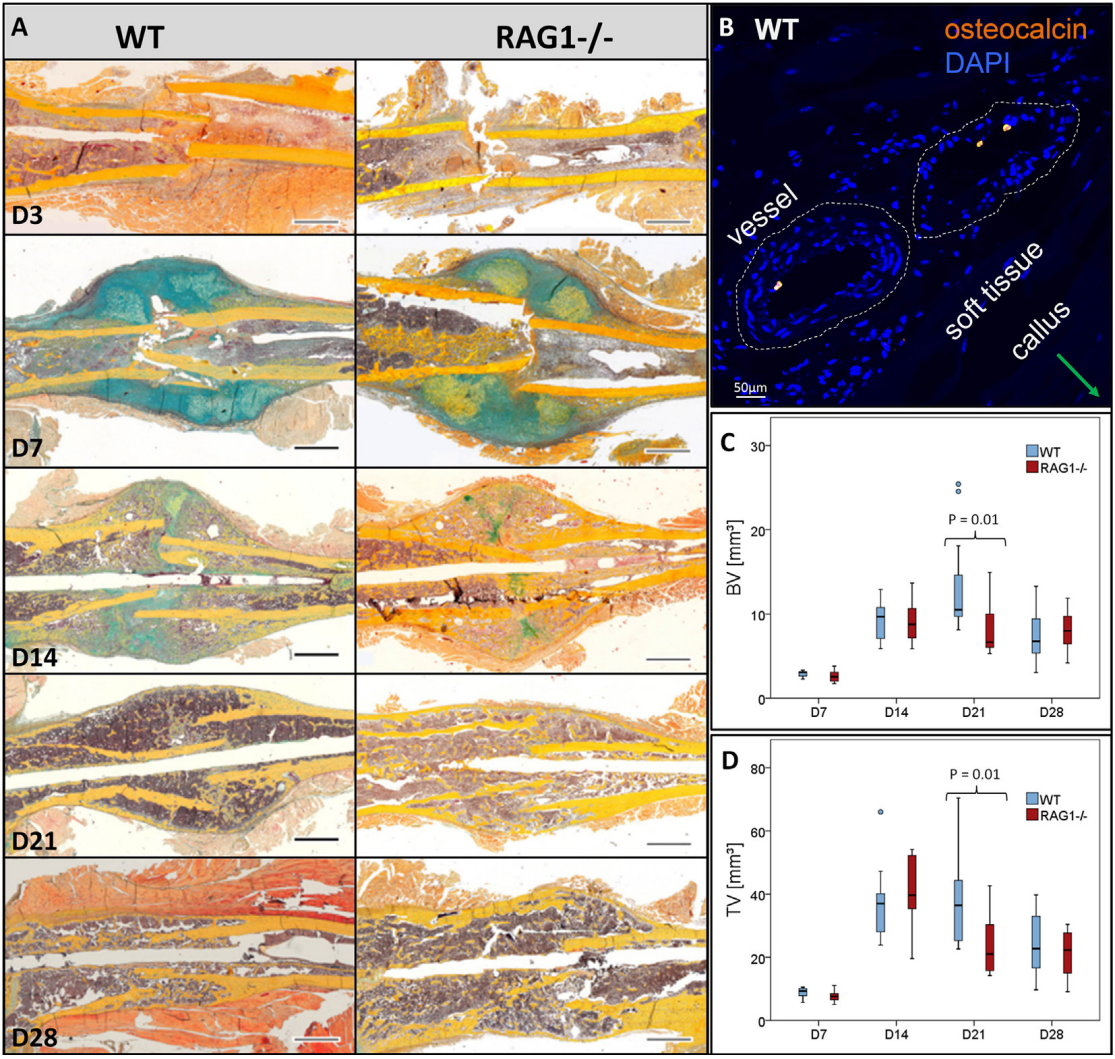
**Fracture healing in wild-type (WT) and recombination activating gene 1 homozygous knockout (RAG1^−/−^) animals: (A) fracture healing in the WT animals showed the typical steps of hematoma [day 3 (D3)], soft callus formation (D7), woven bone deposition (D14), and remodeling (21 and 28 days)**. In the RAG1^−/−^ animals, lack of mature T and B cells has altered this sequence. D3 shows abundant endosteal hematoma, however, periosteal bone formation far from the fracture gap was also seen. 7 days after fracture mineralized batches in the cartilaginous callus indicated enhanced mineralization of the callus, these were more pronounced than in the WT. At D14, the cartilage was vastly calcified and remodeling was enhanced at 21 and 28 days (Movat’s pentachrome staining: bone—yellow, cartilage—blue/green, muscle—red, bone marrow—magenta, and connective tissue— light blue; scale = 1 mm). **(B)** Osteocalcin-positive cells were found in WT in the vessel of the soft tissue surrounding the callus. Micro computed tomography analysis (without normalization to the contralateral bone—to analyze the difference in healing only) showed higher bone volume **(C)** and the total volume **(D)** in the WT animal callus at D21.

The WT showed a typical course of bone healing that consists of consecutive but overlapping phases. The inflammatory reaction that is initiated upon injury and bleeding resulted in visible hematoma formation and maturation (Figure 2, WT, D3). It is followed by cartilaginous callus formation (Figure 2, WT, D7). Upon hypertrophy of chondrocytes the matrix mineralized and the hard callus evolved, which consisted of woven bone (Figure 2, WT, D14). During the final phase of bone healing, remodeling took place thereby slowly rebuilding the form and function of the bone according to its mechanical constraints (Figure 2, WT, D21 and D28). When comparing the bone healing sequence in WT mice and mice lacking mature T and B cells it becomes apparent that ossification seems to be accelerated, which can be seen in the earlier onset of mineralization (Figure 2, RAG1^−/−^, D7). The earlier callus formation in the RAG1^−/−^ animals lacking mature T and B cells has been reported in detail before (4).

During healing, Ocn visualization showed lower signals in the RAG1^−/−^ than in the WT callus at D7 (Figure 2B). Furthermore, microCT analysis of bone healing progression reflected an increased BV and total volume (TV) in the WT animals at D21 (Figures 2C,D, respectively). Both, BV and TV showed a decreasing trend between D21 and D28 in the WT but not in the RAG1^−/−^.

### Bone Deposition Pattern Changes in the Absence of Mature B and T Lymphocytes

To determine how mature T and B cells influence the material formation of bone, we looked into bone apposition during bone healing. Bone formation was monitored using fluorescent dyes injected 2 days prior to sacrifice at 7, 14, and 21 days after fracture to determine new bone formation in the fracture callus with (WT) and without B and T lymphocytes (RAG1^−/−^).

**Figure 3:**
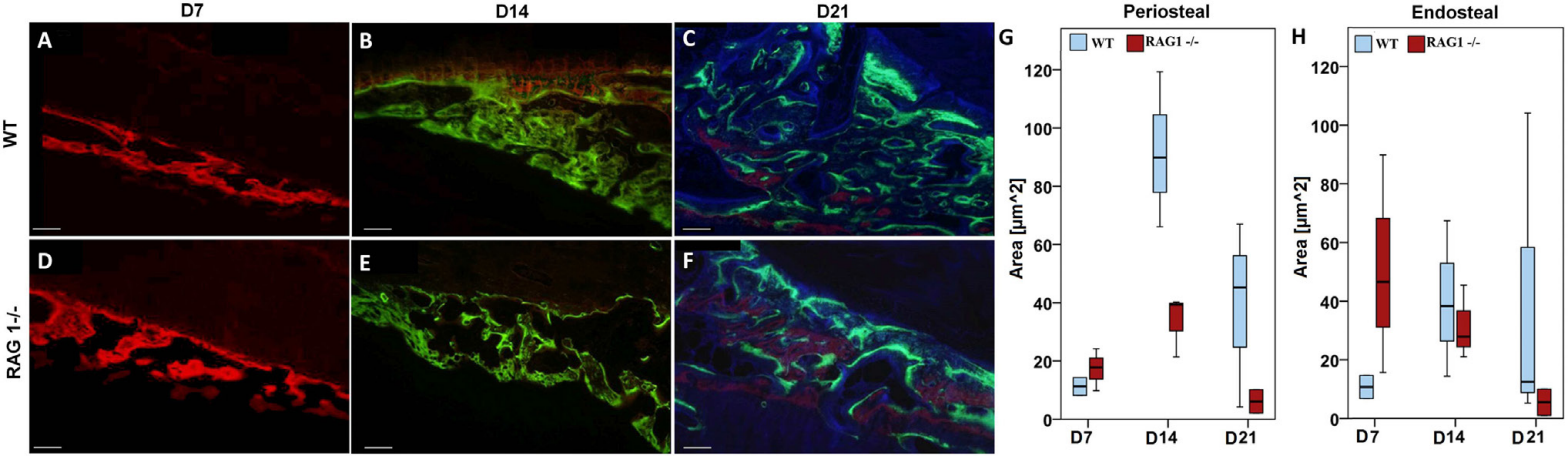
**Fracture callus mineralization differs without mature T and B cells**. Fluorochrome labeling reflects mineral deposition in bone matrix upon binding to a fluorophore. Red labeled areas show mineral deposition at day 7 (D7) [**(A)** for wild-type (WT) and **(D)** for recombination activating gene 1 homozygous knockout (RAG1^−/−^)], green ones reflect deposition at D14 [**(B)** for WT and **(E)** for RAG1^−/−^], these time points show larger deposition areas in the RAG1^−/−^ compared to the WT. However, the area of mineral deposition (blue area) at D21 was larger in the WT **(C)** than in the RAG1^−/−^ **(F)**. Intramembranous/periosteal mineral deposition was higher in the RAG1^−/−^ only at D7 and then higher in the WT **(G,H)**. Interestingly, mineral deposition in the cartilaginous callus/endosteal ossification was not different at D14 or D21, but was higher in the RAG1^−/−^ only at D7. Scale bar: 100 µm, see Table S3 in Supplementary Material for periosteal/endosteal area values (μm^2^) per group and time points.

At D7 post fracture, the RAG1^−/−^ animals showed more bone formation in areas of intramembranous/periosteal ossification (Figures 3A,D,G,H). This result supports the finding of accelerated mechanical stabilization in the absence of mature T and B cells reported earlier (4).

However, this dynamic changed at later healing time points, when WT bone formation exceeded RAG1^−/−^ bone formation at D14 post fracture during the phase of endochondral ossification in normal bone healing (Figures 3B,E,G,H). During the remodeling stage, WT bone formation was distinctively higher than in RAG1^−/−^ bone formation, indicating an active bone forming process in a phase where high numbers of T and B cells are present (Figures 3C,F,G,H) (13). During the remodeling stage, the bone adapts to its mechanical loading that according to “Wolff’s law” determines form and function of the bone.

We conclude that in the presence of T and B cells the bone formation process is guided to allow proper matrix organization leading to distinct bone quality.

### Distinctive Changes in Fibril Fibers in Fracture Callus upon Lack of B and T Lymphocytes

**Figure 4:**
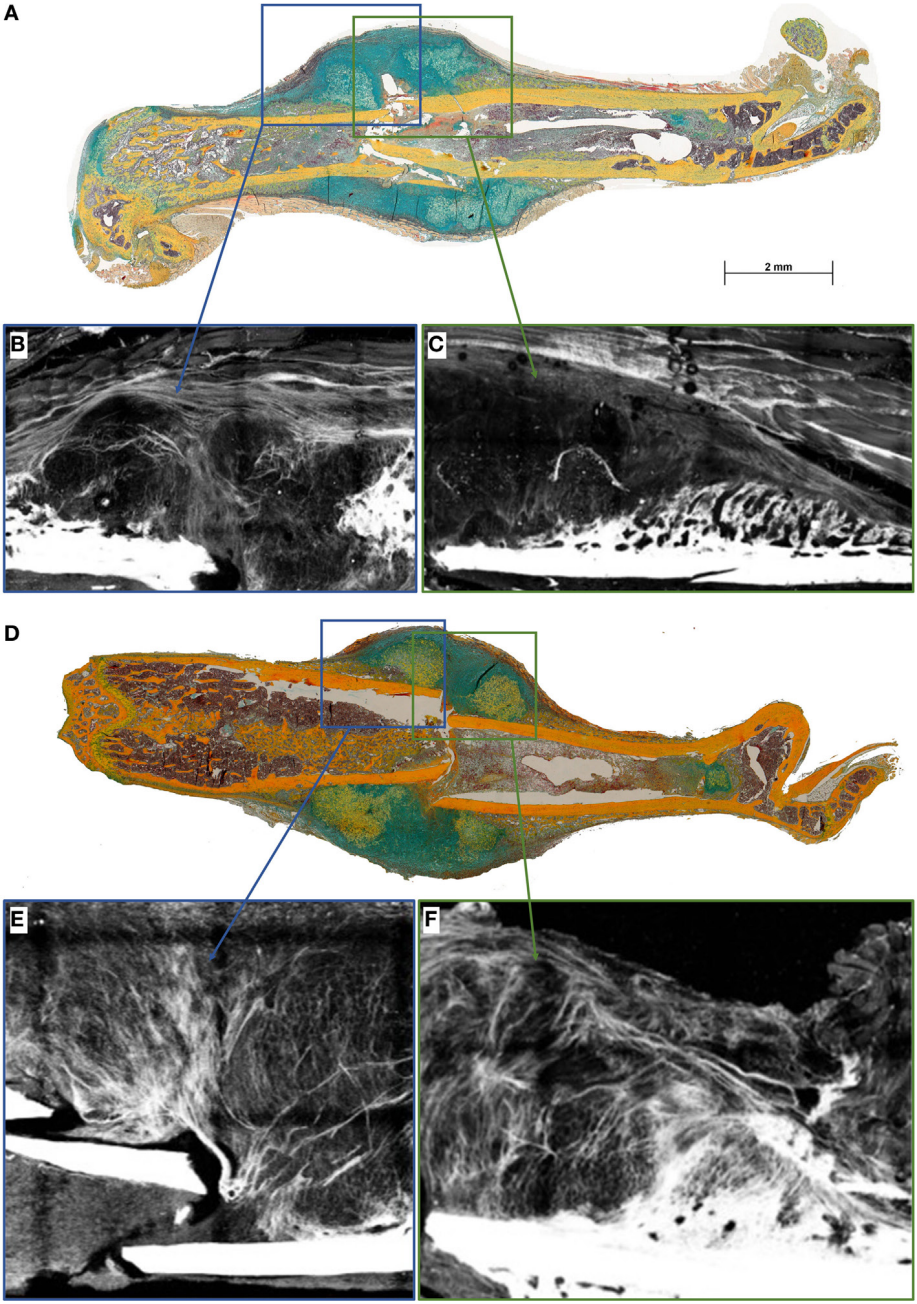
**Collagen fibril deposition appears disorganized in recombination activating gene 1 homozygous knockout (RAG1^−/−^) animals’ callus at day 7 (D7)**. Seven days after fracture the callus was imaged to analyze the collagen deposition pattern in wild-type (WT) mice **(A)** and RAG1^−/−^ mice **(D)**. WT animals show an aligned structure of collagen fibrils **(B)** in the callus and on the periosteal surface **(C)**, the imaging also shows structurally sound woven bone on the periosteal surface. Disarrangement of collagen fibrils structure was apparent in the RAG1^−/−^ callus **(E)**, and periosteal bundle arrangement is seen with dense mineral deposition in the periosteal surface without woven bone structure **(F)**. This representative image shows the disorganized collagen deposition occurring without mature T and B cells in the RAG1^−/−^ animals. (Blue rectangles show the central area of the fracture callus while the green outtakes concentrate on the margin and thus periosteal callus area.)

To further unravel the impact on matrix formation in animals lacking mature T and B cells, we investigated fibrillar collagen fiber deposition during bone healing in the early fracture callus using second harmonic photon microscopy (D7 post fracture). While fibers in the WT seem to be aligned along the outer callus border and also surrounding the dark areas that would indicate cartilaginous zones, this was not found in the RAG1^−/−^ calluses (Figure 4).

**Figure 5:**
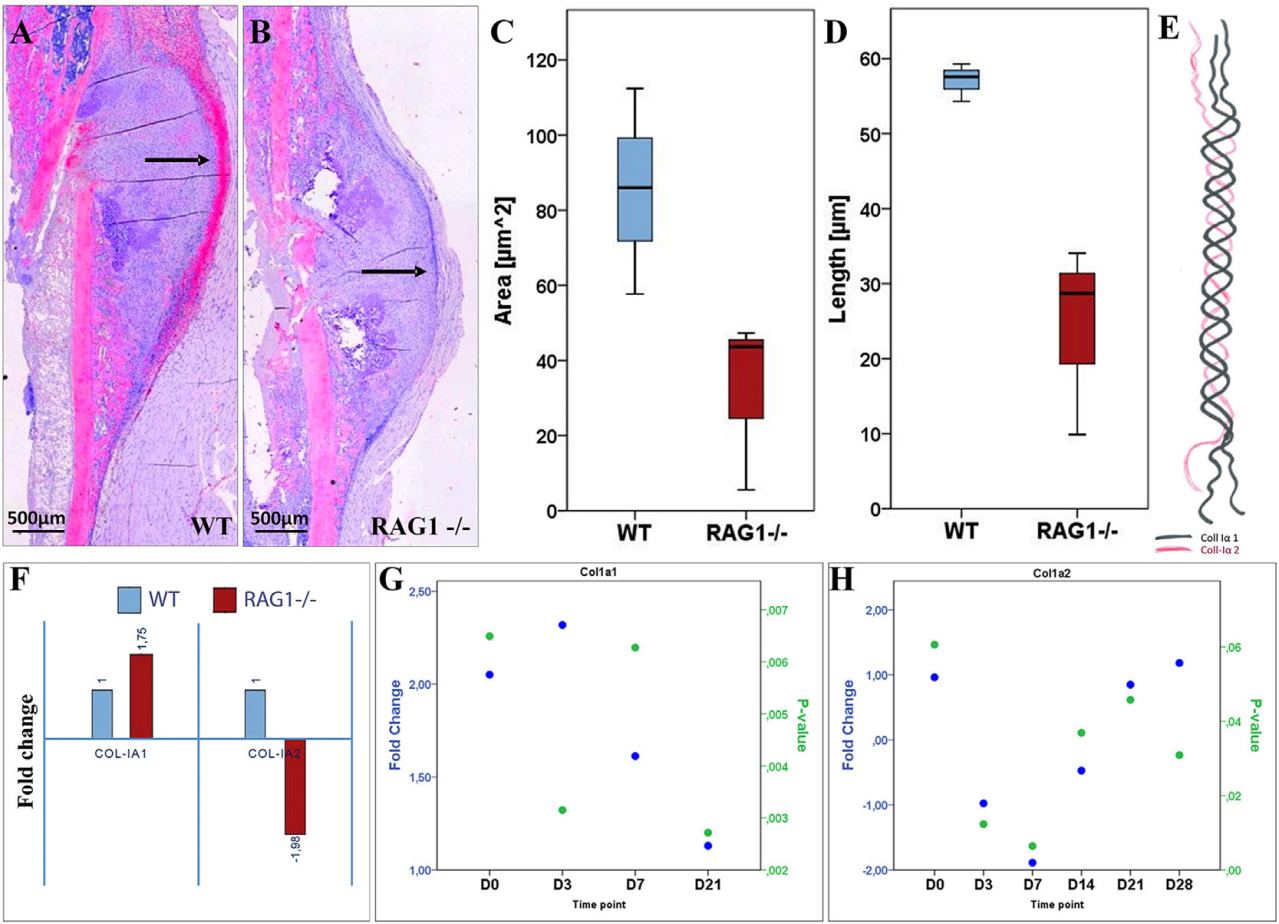
**Collagen type I (ColI) deposition and expression pattern are considerably altered in fracture healing when mature T and B cells are absent**. **(A)** Histological staining of ColI (2.5× overview) shows higher signal intensity (indicated by black arrows) on the periosteal surface and the callus outer borders in the wild-type (WT) at day 7 (D7). **(B)** Lack of signal location at the callus outer border in the recombination activating gene 1 homozygous knockout (RAG1^−/−^) at D7 with general lower signal intensity. Compared to the RAG1^−/−^, larger ColI-positive area in the WT **(C)** and longer surface in the WT **(D)** are detected. **(E)** ColI composition: two subunits of ColIα1 and one subunit of ColIα2 form the typical ColI triple helix. **(F)** The expression of the ColI subunits Iα1 and Iα2 at D7 shows a 1.75-fold increase in ColIα1, while the expression of ColIα2 was decreased by 2. Changed expression patterns for ColIα1 **(G)** and ColIα2 **(H)** were observed throughout the observed healing period of 28 days.

For bone formation, ColI deposition proceeds with the binding of hydroxyapatite during matrix mineralization. Since bone formation was affected in RAG1^−/−^, ColI was analyzed during bone healing in both animal types by immunohistochemical staining. In the WT animals, a typical outer callus area was positive for ColI, while this ColI-positive area was missing in the RAG1^−/−^ animals (Figures 5A–D).

ColI is a fiber composed of two ColIα1 and one ColIα2 fibrils (Figure 5E). Upon analyzing the expression of ColI subunits, a significantly altered expression pattern was detected (Figures 5G,H; see Table S2 in Supplementary Material). Interestingly, at D7 ColIα1 expression was nearly two times higher in the RAG1^−/−^, while ColIα2 was nearly two times lower when compared to the WT group (Figure 5F). This indicates a severe dysregulation of both subunits of ColI, which represents the most important matrix component of bone. Without mature T and B cells, the synthesis and deposition of ColI were distinctly changed during the early fracture healing stages.

### Osteoblast Cell Dynamics Are Distinctly Altered without Mature T and B Cells

**Figure 6:**
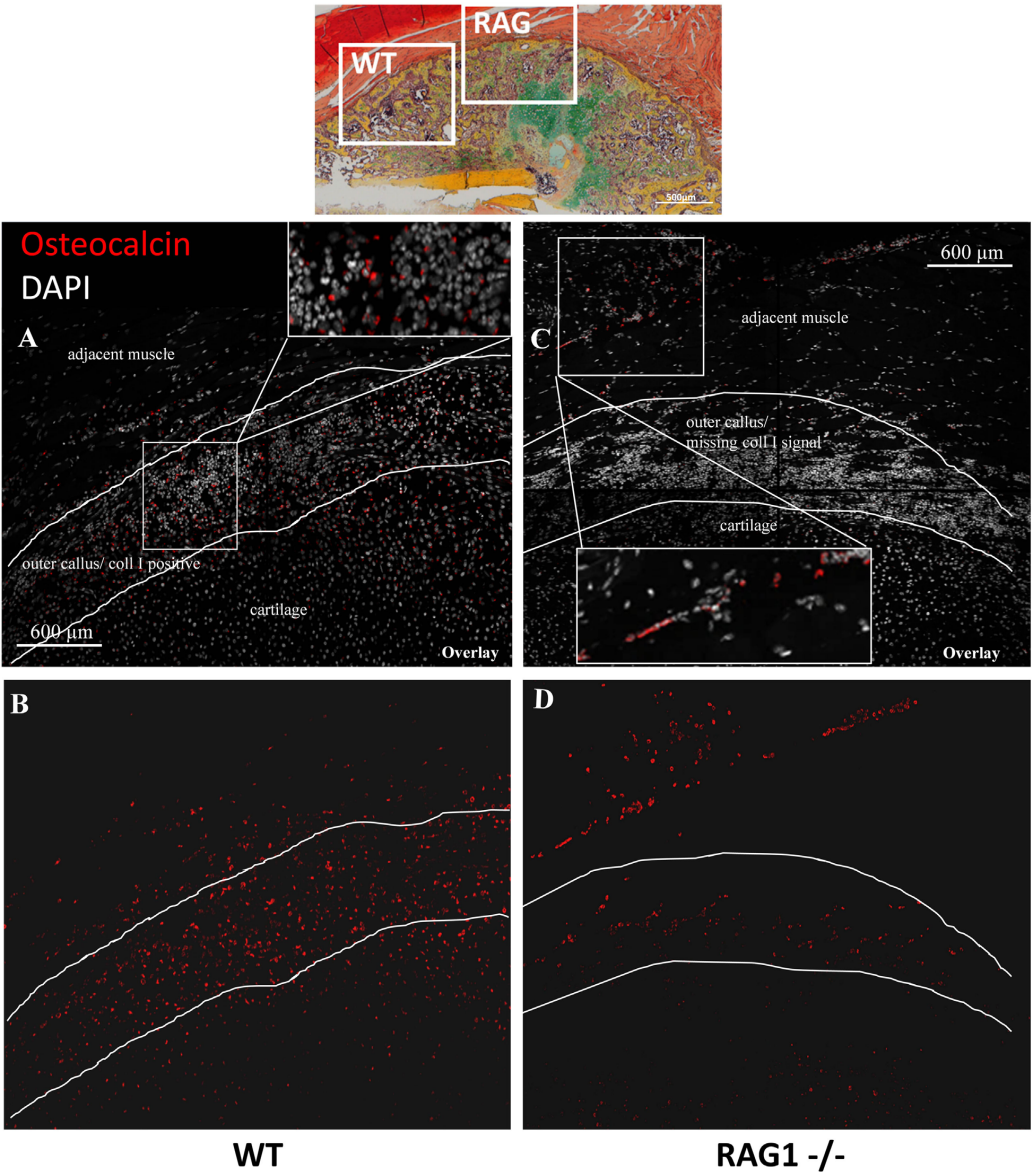
**Osteocalcin (Ocn)-positive cells show an aberrant localization in the fracture callus of animals healing without mature T and B cells**. Immunohistological images of Ocn-positive cells in the fracture callus of WT **(A,B)** and recombination activating gene 1 homozygous knockout (RAG1^−/−^) animals **(C,D)** at day 14 reveal an abundance of these cells in the collagen type I-positive outer rim of the wild-type callus **(A)** while these cells are scarce in the equivalent location in RAG1^−/−^ animals **(C)**. In the RAG1^−/−^ animals, a layer of Ocn-positive cells appears in the soft tissue adjacent to the fracture callus (red = Ocn; gray = DAPI to stain cell nuclei).

With the clear evidence that the lack of mature T and B cells leads to extracellular matrix (ECM) alteration in bone tissues during organogenesis and very pronounced in bone healing, the cells responsible for ColI production were analyzed. ColI is mainly produced by osteoblasts during the bone formation process. Due to the altered ColI pattern in RAG1^−/−^ mice, histological sections were analyzed for Ocn-positive osteoblasts (Figure 6). In WT animals, osteoblasts were concentrated in the cell rich outer callus layer jointly with fibrillar fibers and an intense ColI expression (Figures 6A,B). In RAG1^−/−^ animals, Ocn-positive cells were concentrated in a distinctly different area, in the connective tissue and muscle layer directly adjacent to the fracture callus but not at the forefront of callus formation (Figures 6C,D).

The abovementioned data suggested that T and B cells are important for the tissue properties of newly formed bone material and that osteoblasts are located distant to the callus tissue formation front if mature T and B cells are missing. Thus, the interdependency of the immune cells and the bone formation signals were analyzed during fracture healing using expression analysis.

A detailed examination of osteoclasts, bone cells responsible for bone degradation revealed no significant difference in osteoclasts numbers in the healing cascade up to D14. At D14, RAG1^−/−^ showed significantly more osteoclasts (*p* = 0.037): at this time point occurs typically the maturation from the soft callus to hard callus and the beginning woven bone deposition in WT animals (Figure S2 in Supplementary Material).

### Expression Analysis Confirms That the Changes in RAG1^−/−^ Bone Formation Are Linked to Mature T and B Cells

**Figure 7:**
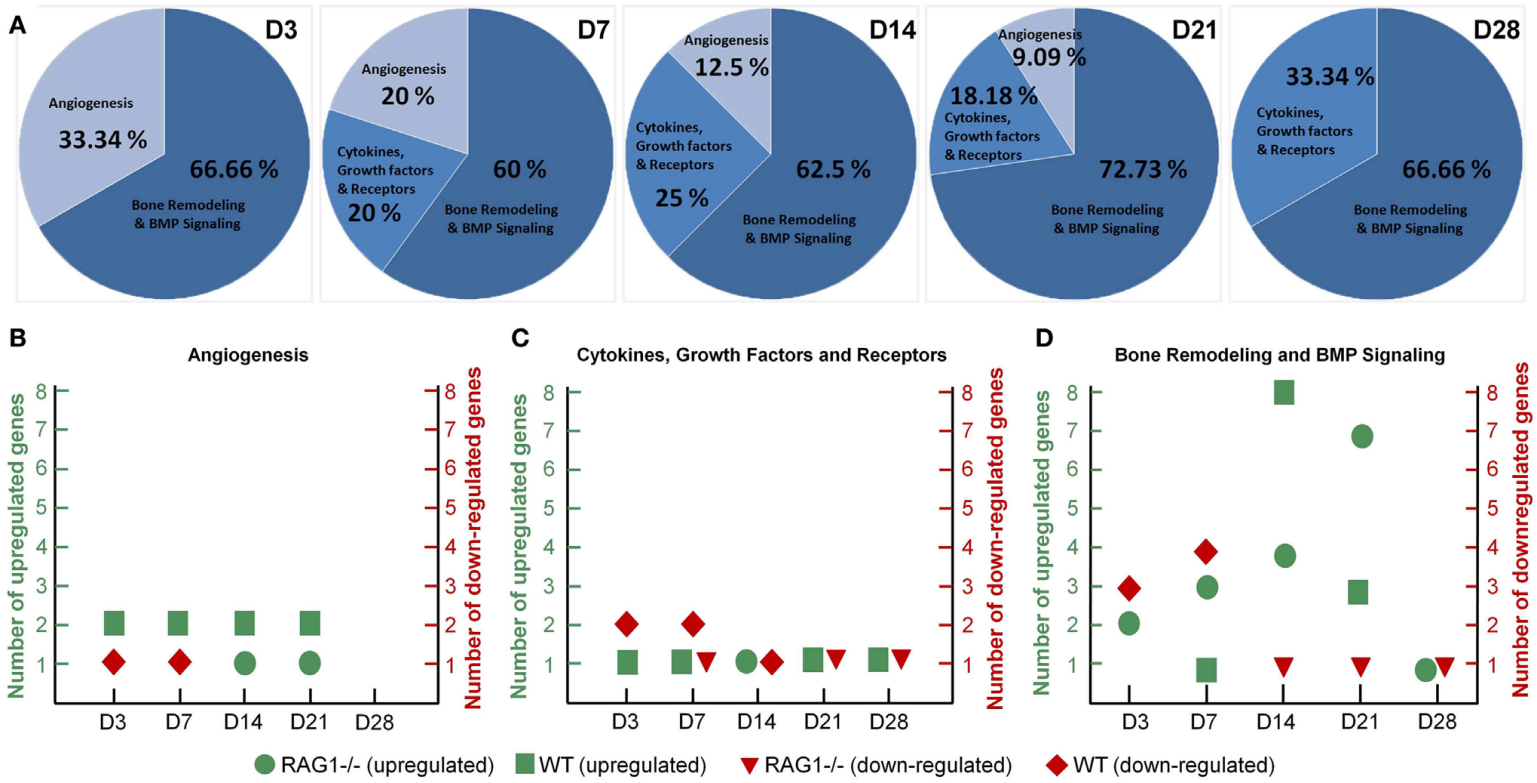
**Dysregulated angiogenesis and cytokine expression in the recombination activating gene 1 homozygous knockout (RAG1^−/−^) callus along with chronically shifted (earlier) bone formation and remodeling**. **(A)** Angiogenesis-related genes showed declining numbers of differentially expressed genes with healing progression in the RAG1^−/−^. Cytokines and growth factor gene expression was highest at day 28 (D28). Interestingly, the percentage of bone remodeling and formation differentially expressed genes was almost constant throughout the healing in the RAG1^−/−^ fracture callus. **(B)** Mostly, wild-type (WT) genes were upregulated at all time points, whereas upregulation in the RAG1^−/−^ was only at D14 and D21. **(C)** RAG1^−/−^ genes were upregulated only at D14. **(D)** Number of upregulated genes in the RAG1^−/−^ increased from D3 to D14, however, at D28 upregulated genes were still seen in the RAG1^−/−^.

To identify the discrepancies in bone healing we have limited the comparison of RAG1^−/−^ and the WT callus tissue to the gene sets selected in commercial array panels for angiogenesis, cytokines, and growth factors, and bone remodeling and BMP (bone morphogenetic protein) signaling (Qiagen, Hilden, Germany). In RAG1^−/−^ mice, when D0 was taken as control to look into the changes during the bone healing process, the gene list was compared to the panels for an overview of the change in expression. At D3, over 30% of the differentially expressed genes were related to angiogenesis (Figure 7A), the percentage declined from D7 (20%) to D14 (12.5%) to D21 (9%) to 0% at D28. However, the cytokine- and growth factor-related genes showed an irregular pattern with 0 differentially expressed genes at D3, an increase from 20% at D7 to 25% at D14 followed by a decrease at D21 (18%) and again an increase at D28 (33%) (Figure 7A). Interestingly, the panel involving bone remodeling and BMP signaling (Figure 7A) showed an approximate consistency at all time points from D3 (67%), D7 (60%) through D14 (62.5%), D21 (73%) until D28 (67%).

Additionally, when the RAG1^−/−^ group was compared to the WT group, more genes were seen to be upregulated in the WT group in the angiogenesis panel, whereas genes in the RAG1^−/−^ group on the other side were downregulated at D3 and D7 and upregulated only at D14 and D21 (Figure 7B). However, cytokines and growth factors panel showed that more genes were downregulated in the WT group at D3 and D7, but upregulated at all time points in comparison with the RAG1^−/−^ mice (which showed a downregulation of genes at D7, D21, and D28 and an upregulation only at D14) (Figure 7C). In the bone remodeling and BMP signaling panel, RAG1^−/−^ mice showed differentially expressed genes at all the time points with the highest number of upregulated genes at D21, while WT showed the highest number of upregulated genes at D14 when compared to RAG1^−/−^ (Figure 7D). The gene number blot (Figure 7D) showed a shift in upregulation/downregulation patterns in genes related to bone remodeling between the two groups.

### The Effect of Changed Healing Pattern in the RAG1^−/−^ Animals Is Predominantly Caused by the Lack of T Cells

**Figure 8:**
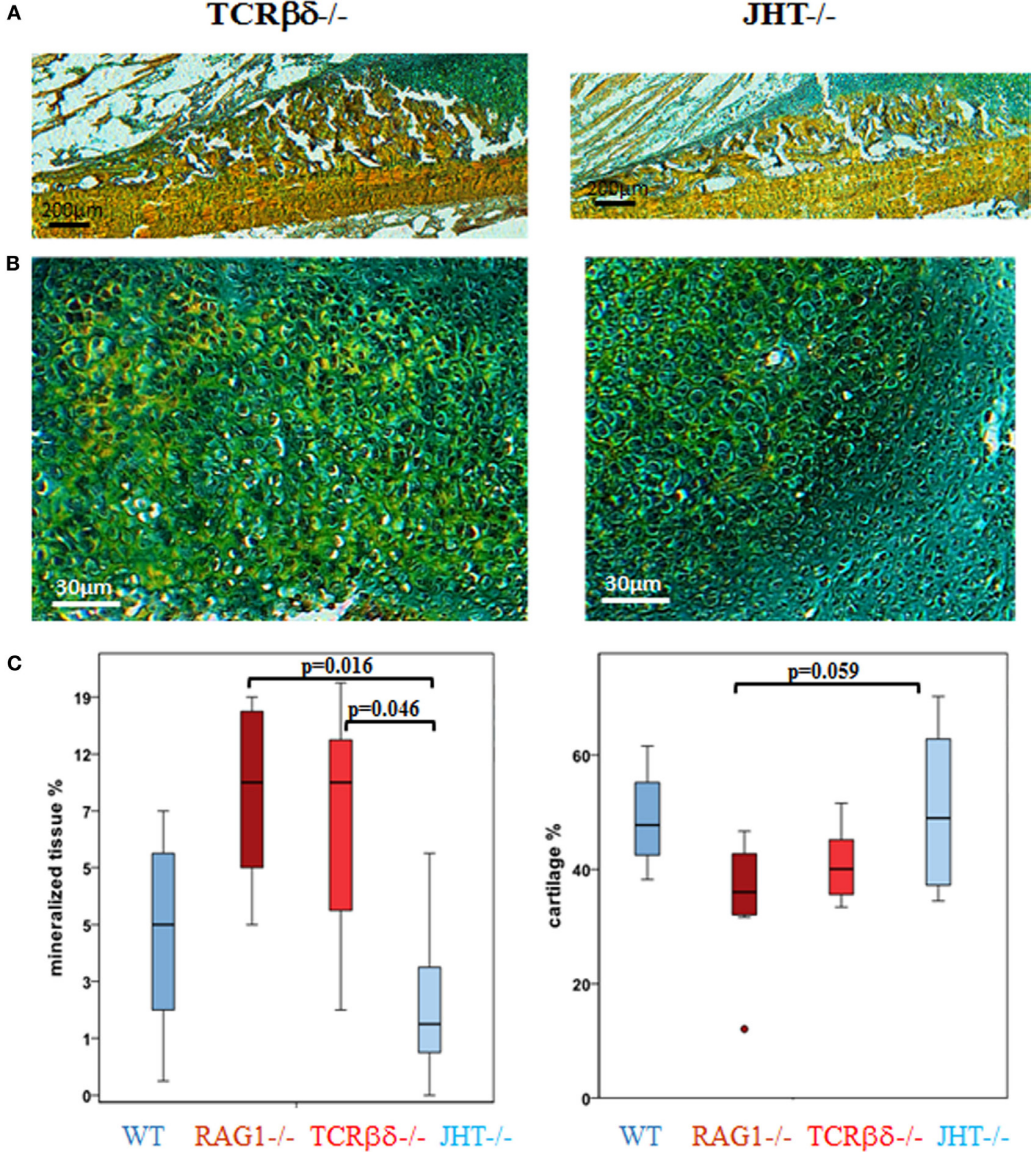
**Single knockouts for either T cells or B cells reveal a distinct healing pattern**. In T cell-deficient mice healing resembles recombination activating gene 1 homozygous knockout (RAG1^−/−^) animals, while B cell-deficient mice resemble the wild-type (WT) healing more closely. Movat’s pentachrome staining of fracture healing 7 days into healing revealed an early mineralization in T [T cell receptor β and δ chain homozygous knockout (TCRβδ^−/−^)] and B [joining region homozygous knockout (JHT^−/−^)] cell-deficient mice **(A,B)** that was also seen in RAG1^−/−^ animals and which exceeds the mineral deposition in B cell-deficient mice. **(C)** A histomorphometrical analysis of the bone and cartilage tissue in the fracture callus confirmed the similarity in RAG1^−/−^ and T cell-deficient mice (red) when compared with WT healing and the healing in B cell-deficient mice (blue).

Changes in mineralization in RAG1^−/−^ could be dependent on the lack of T cells or B cells independently. Therefore, the 7-day healing process was assessed in animals that lacked either T cells (TCRβδ^−/−^) or B cells (JHT^−/−^). The histological images showed that in the animals that lacked only T cells an earlier mineralization was detected (Figures 8A,B, left) and the cartilaginous area showed signs of mineralized matrix earlier as well (Figure 8C).

Histomorphometrical analysis showed that mineralized values were similar in RAG1^−/−^ and T cell devoid healing (Figure 8C, left). In conclusion, B cell devoid healing was similar to WT healing, while animals without T cells showed healing patterns similar to the one in RAG1^−/−^ animals. Both groups, RAG1^−/−^ and T cell knockout animals, showed significantly more mineralized tissue in comparison to the B cell knockout group. A similar pattern was found upon analyzing the cartilaginous areas (Figure 8C, right). These findings support the assumption that the changes in the bone quality seen in the RAG1^−/−^ bone healing are primarily due to the lack of T cells.

### T Cells Drive Normal Deposition of ColI during Fracture Healing

Due to the striking difference in mineralization capacity between mice specifically devoid of either T or B cells, we decided to further analyze bone healing in these animals at the cellular level by confocal microscopy.

**Figure 9:**
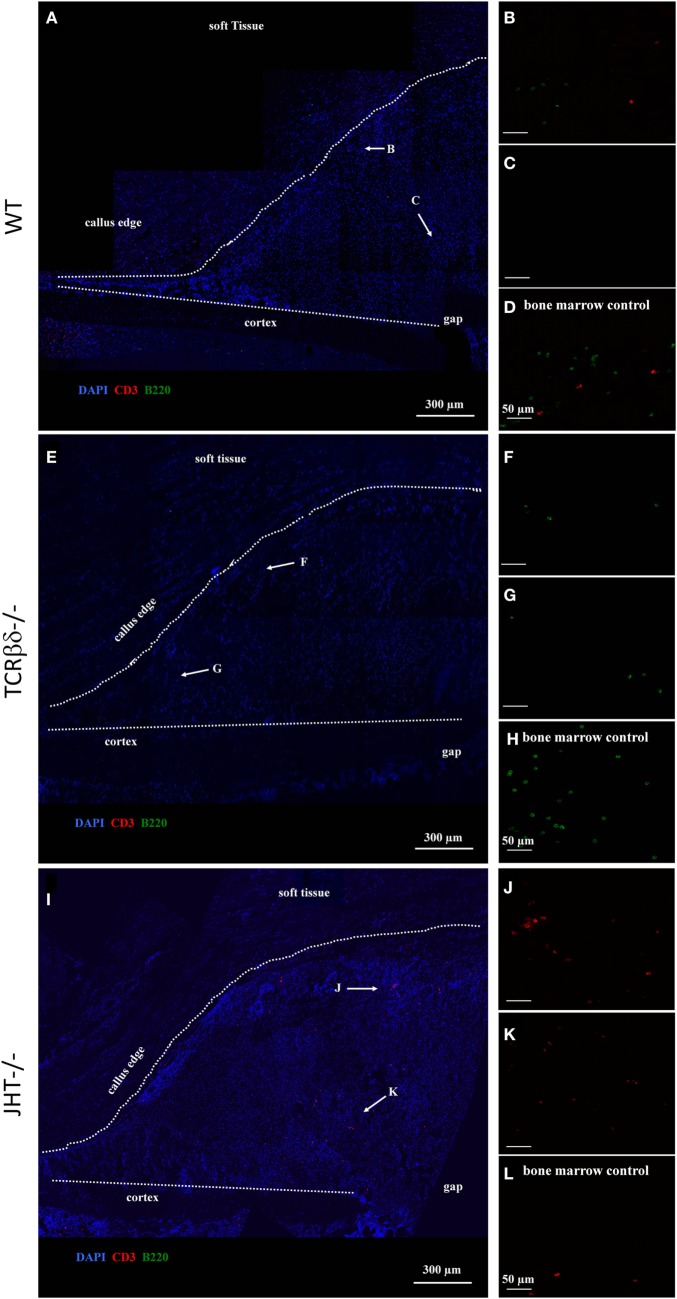
**Altered migration pattern of T cells in the early callus of B cell-deficient mice**. Confocal microscope analysis of the callus of wild-type (WT) **(A–D)**, T cell-deficient [T cell receptor β and δ chain homozygous knockout (TCRβδ^−/−^)] **(E–H)** and B cell-deficient [joining region homozygous knockout (JHT^−/−^)] **(I–L)** mice. Sections were stained for the T cell marker CD3 (red), B cell marker B220 (green), and cell nuclei (DAPI, blue). Letters near the white arrows indicate areas that are magnified in the corresponding panels below **(A,E,I)**. **(D,H,L)** Control staining in the bone marrow of WT, T cell—and B cell—mice, respectively. All sections were taken 7 days after fracture.

At 7 days post fracture and similar to controls, small numbers of B cells were detected in the callus periphery of T cell-deficient mice (Figures 9E–G). On the other hand, T cell migration was altered in the callus of B cell-deficient mice (Figures 9I–K). In fact, as previously described (13), both T and B cells normally reside in the outer callus edge and not in the inner areas at this time point (Figures 9A–C). However, T cells infiltrated in large numbers in the inner callus of B cell-deficient mice, indicating a clear modification of their migratory activity. Control staining in the bone marrow of WT, T cell and B cell deficient mice, respectively (Figures 9D,H,L).

**Figure 10:**
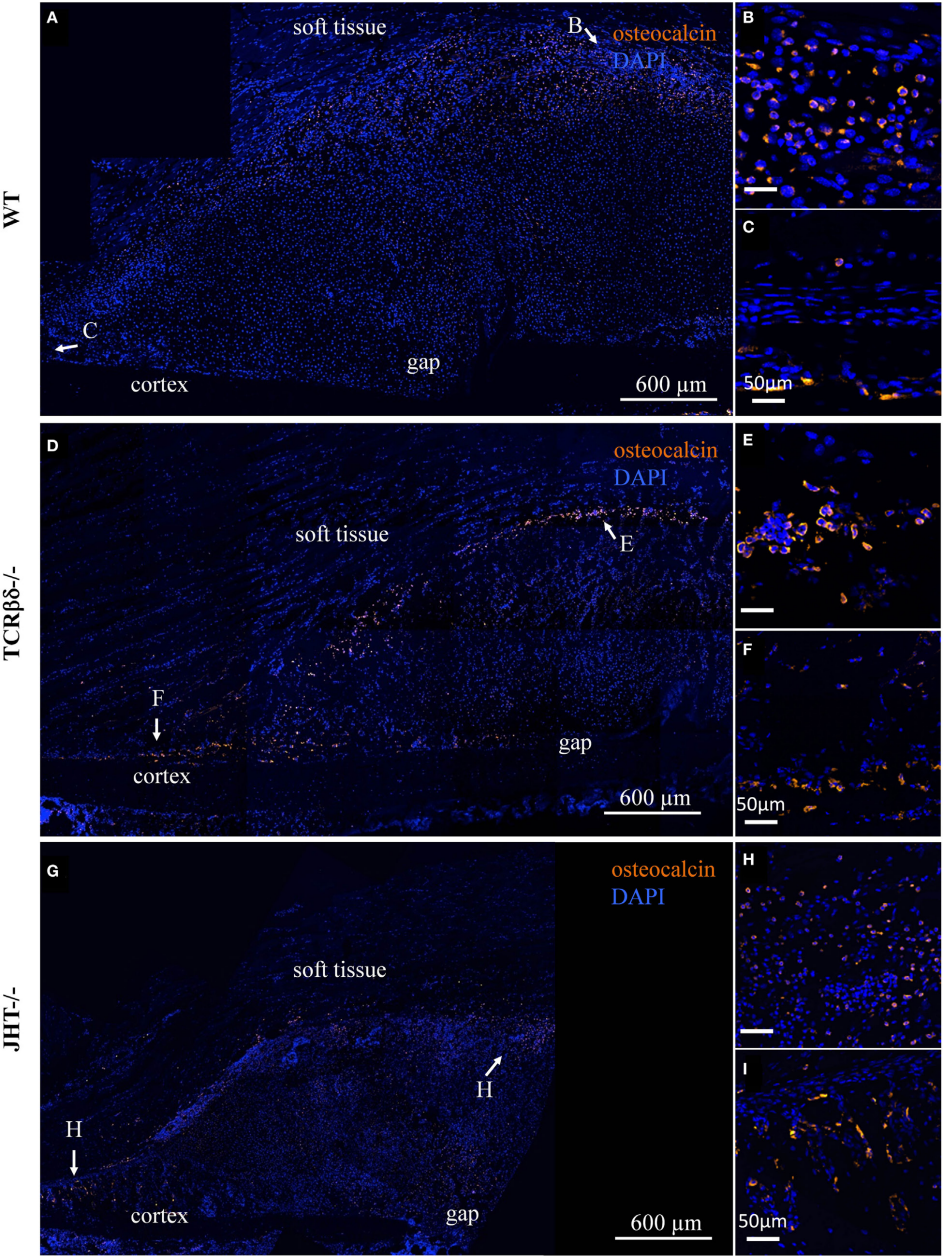
**Infiltration of the soft callus by osteoblast precursors in lymphopenic mice**. Confocal microscope analysis of the callus of wild-type (WT) **(A)**, T cell-deficient mice [T cell receptor β and δ chain homozygous knockout (TCRβδ^−/−^)] **(D)** and B cell-deficient [joining region homozygous knockout (JHT^−/−^)] **(G)** mice. Sections were stained for the osteoblast marker osteocalcin (orange) and cell nuclei (DAPI, blue). Letters near the white arrows indicate areas that are magnified in the corresponding panels to the right **(B,C,E,F,H,I)**. All sections were taken 7 days after fracture.

In order to test whether this change in lymphocyte migration also influenced the redistribution of cells of the osteoblast lineage in the callus, we stained adjacent sections for Ocn. Interestingly, no evident change in osteoblastic cell migration was visible in either T cell- or B cell-deficient mice (Figure 10).

**Figure 11:**
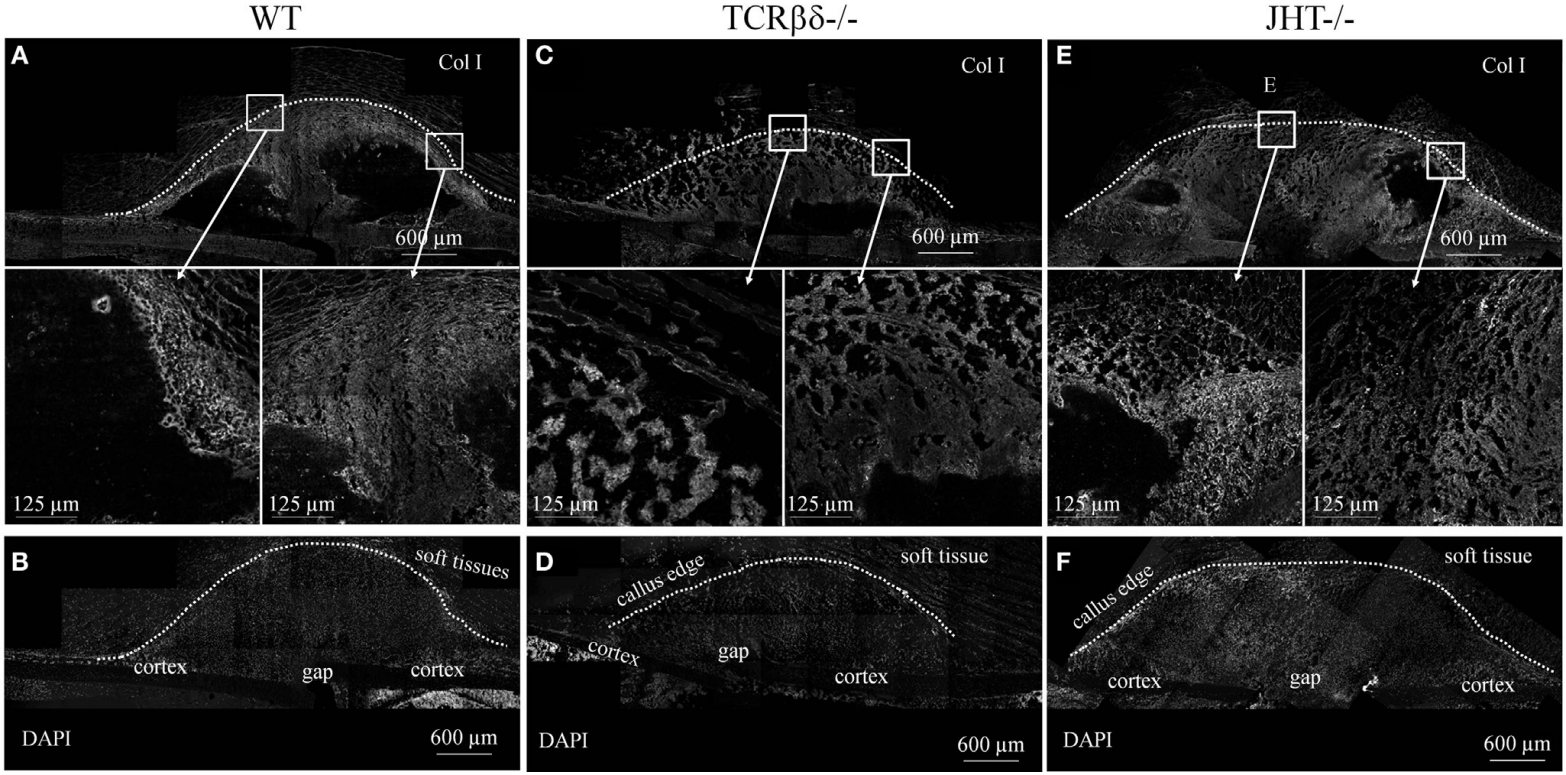
**Aberrant deposition of collagen type I (ColI) in the soft callus of T cell-deficient mice**. Confocal microscope analysis of the callus of wild-type (WT) **(A,B)**, T cell-deficient [T cell receptor β and δ chain homozygous knockout (TCRβδ^−/−^)] **(C,D)** and B cell-deficient [joining region homozygous knockout (JHT^−/−^)] **(E,F)** mice. Sections were stained for the ColI (gray). Areas within the white rectangles are represented below in a higher magnification. **(B,D,F)** show DAPI staining of cell nuclei to highlight the cellular topography corresponding to calluses displayed in **(A,C,E)**, respectively. Soft tissues are visible due to their strong autofluorescence. All sections were taken 7 days after fracture.

This, however, could not exclude that osteoblastic function was altered in the groups of mice analyzed. In order to test this hypothesis, we stained overlapping sections for ColI. An aberrant pattern of ColI deposition was detected in T cell-deficient mice (Figures 11C,D), which strongly resembled what we observed in RAG1^−/−^ mice (Figures 4 and 5). In fact, large areas of the callus were devoid of ColI in T cell-deficient mice, giving it a beehive-like appearance (Figures 11C,D). Furthermore, collagen did not form the characteristic thick arch of fibers that bridges the cortices across the gap (Figures 11A,B). Similar to what was detected using SHG microscopy on RAG1^−/−^ mice fractures (Figure 4), the collagen was not deposited across the callus parallel to its edge in T cell-deficient mice, but rather disorderly all over the surface (Figures 11C,D).

On the other hand, B cell-deficient mice showed similar expression patterns in immunohistochemistry to WT controls (Figures 11E,F). However, large amounts of ColI were detected in the inner areas of the callus where T cells were found to abnormally infiltrate at 7 days after fracture (Figures 11E,F and 9I–L).

**Figure 12:**
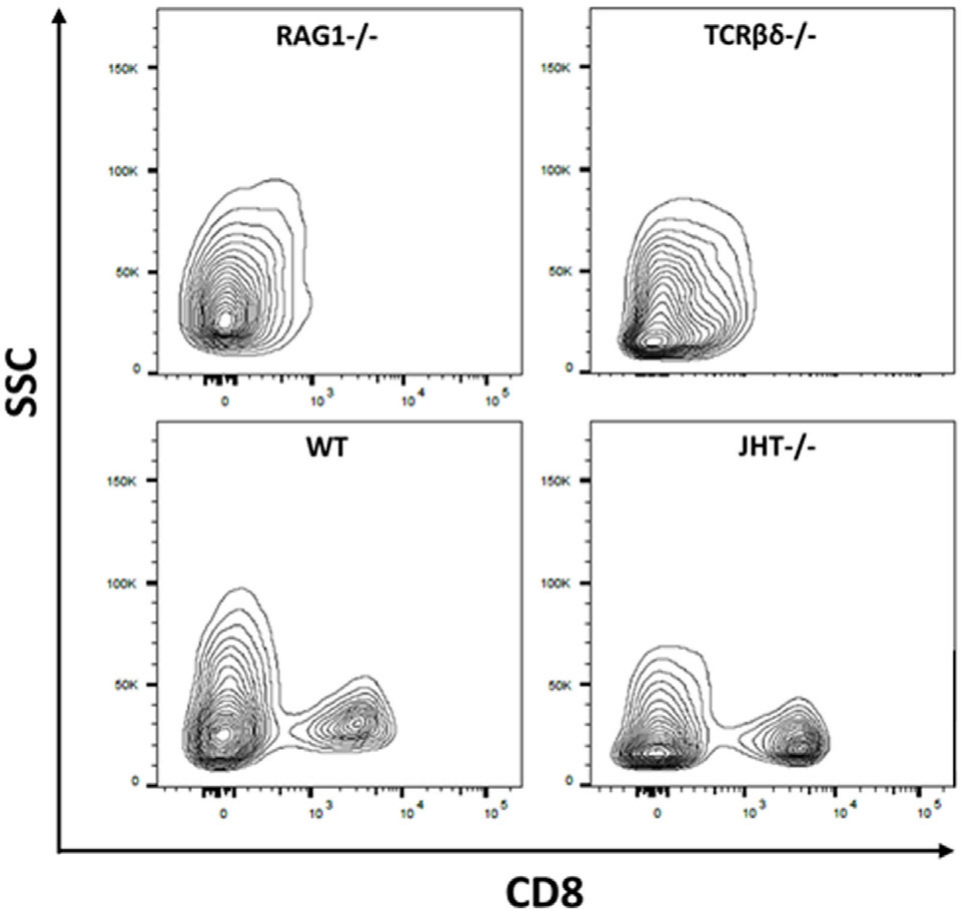
**Cellular composition of wild-type (WT), recombination activating gene 1 homozygous knockout (RAG1^−/−^), T cell receptor β and δ chain homozygous knockout (TCRβδ^−/−^), and joining region homozygous knockout (JHT^−/−^) mice**. Flow cytometry was used to determine whether CD8^+^ T cells are present in the mouse strains used in this analysis. CD8^+^ T cells have been shown to impact bone healing (7). While RAG1^−/−^ and TCRβδ^−/−^ lacked CD8^+^ T cells these cells were present in WT and JHT^−/−^ animals. Gating: lymphocytes, single cells, live cells, CD45-positive cells, CD3-positive cells, and CD8-positive cells.

**Figure 13:**
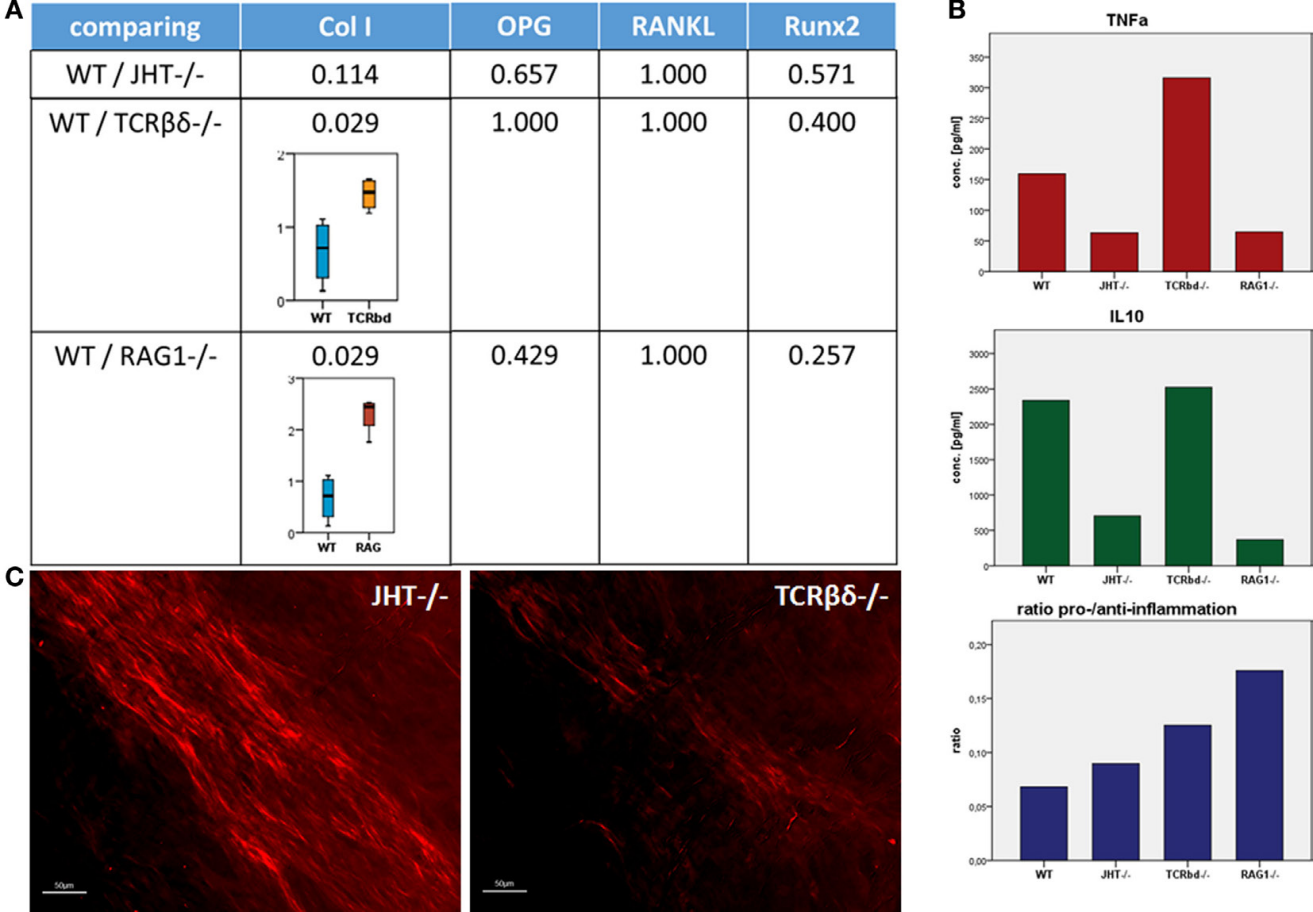
**Collagen I deposition is irregular in recombination activating gene 1 homozygous knockout (RAG1^−/−^) and T cell receptor β and δ chain homozygous knockout (TCRβδ^−/−^) mice**. **(A)** Using quantitative reverse transcription polymerase chain reaction a significant difference in expression of collagen I was detected in bone marrow cells of RAG1^−/−^ and TCRβδ^−/−^ mice when compared to wild-type (WT). Bone markers such as osteoprotegerin (OPG), receptor activator of NF-kB ligand (RANKL), and Runt-related transcription factor 2 (RUNX2), however, were not affected. **(B)** Splenocytes were activated with LPS, and conditioned medium was analyzed to determine pro-inflammatory (TNFα) and anti-inflammatory (interleukin 10) cytokines. While these factors showed no correlation in itself, the ratio of pro- and anti-inflammatory signals showed higher pro-inflammatory signaling in RAG1^−/−^ and TCRβδ^−/−^ when compared to WT and JHT^−/−^. This indicates an influence of the cytokine pattern of T and B cells on the bone forming process. **(C)** Conditioned medium was added in an *in vitro* assay of mesenchymal stem cells (MSCs), and collagen deposition was analyzed using a Sirius red staining and polarization microscopy. Collagen deposition by MSCs cultivated with conditioned medium of JHT^−/−^ splenocytes showed a stronger and more regular orientation than collagen deposition of MSCs cultivated with conditioned medium of TCRβδ^−/−^ splenocytes.

To understand the mechanism behind the influence of the T and B cells on the collagen I deposition process, we confirmed the presence of CD8^+^ T cells in the mouse models (WT, RAG1^−/−^, TCRβδ^−/−^, and JHT^−/−^). CD8^+^ T cells are present in WT and JHT animals, while they are missing in RAG1^−/−^ and TCRβδ^−/−^ animals (Figure 12). The collagen apposition is irregular in the absence of T cells and the mRNA analysis via RT-qPCR underline this abnormality in the absence of T cells (TCRβδ−/− and RAG1−/−) but not in the absence of B cells (JHT^−/−^) in the bone marrow. Significant difference in expression of collagen I was detected in bone marrow cells of RAG1^−/−^ and TCRβδ−/− mice when compared to wild-type (WT). Bone markers such as osteoprotegerin (OPG), receptor activator of NF-kB ligand (RANKL), and Runt-related transcription factor 2 (RUNX2), however, were not affected (Figure 13A). CD8^+^ T cells have previously been determined to have an essential impact on the bone healing process, especially in view of TNFα producers (7). Another cytokine, IL10 has been shown as an enhancer of bone healing in an anti-inflammatory context (12). These two cytokines were analyzed in conditioned medium gained from LPS-activated splenocytes of WT, RAG1^−/−^, TCRβδ^−/−^, and JHT^−/−^ animals. Analyzing the protein expression with an ELISA assay showed differential protein expression throughout the mouse models, however, the ratio of TNFα and IL10 showed a more pro-inflammatory cytokine composition for RAG1^−/−^ and TCRβδ^−/−^ (Figure 13B). Even though this is only a fraction of the cytokine pattern that guides bone formation it could be an indication of the mechanism behind the changed collagen I deposition. To verify this, we cultivated MSCs with the conditioned medium won from JHT^−/−^ and TCRβδ^−/−^ splenocytes, respectively. Collagen deposition was analyzed using Sirius red staining and polarization microscopy. More collagen was deposited by MSCs cultivated with conditioned medium from JHT^−/−^ splenocytes and this collagen showed a higher orientation when compared with the collagen formation under TCRβδ^−/−^ conditioned medium (Figure 13C). These *in vitro* results are in accordance with the above shown *in vivo* results.

Overall, these data suggest that B cells can influence the migratory activity of T cells in the callus and that the latter lymphocytes are essential for normal deposition of ColI in the early stages of fracture healing.

## Discussion

Bone healing developed over thousands of years of evolution to a unique and highly successful process of complete regeneration. The faster stabilization observed in mice without mature T and B cells now raises the question on the role of the adaptive immune system in bone healing. A superior healing without adaptive immunity seems unlikely as it has evolved within nature’s selective evolutionary process. Therefore, we aimed to understand the distinct role adaptive immunity plays in the healing cascade. Why would the apparently retarded immune-mediated healing process be beneficial over the faster healing in the absence of T and B cells? The major changes detected during the healing process without mature T and B cells concerned the faster cartilage mineralization. The deposition of cartilage to bridge the fractured bone offers at least three advantages. First, cartilage can be quickly synthesized to bridge the fracture. Second, —the intermediate cartilage tissue offers the possibility to increase the radius of the bone in the area of injury. Considering the fact that stability increases with the fourth power of the radius increase (28), this is a significant advantage. This phenomenon can be observed in the cases of insufficient fracture stabilization, where a larger callus develops when compared to a stable fixed fracture (29)—an attempt of the body to provide stability. Third, the mechanical properties of the proteins in cartilage tissue. Type II collagen offers tensile strength, while proteoglycans and ECM provide compressive strength (30)—together resulting in a fast recovery of function of the injured bone. For bone healing, the important step of intermediate cartilage formation is still intact in lymphopenic RAG1^−/−^ mice. However, in these animals the subsequent degradation and mineralization of the cartilage were altered, with accelerated mineralization visible at D7 of healing, whereas, in the WT, hyaline cartilage was still predominant (Figure 2). The same faster kinetics was seen in animals lacking T cells but not in those devoid of only B cells. Thus, this discussion concentrates on the effect of T cells in the bone healing process.

Previous studies on fracture healing in lymphopenic RAG1^−/−^ mice showed a faster callus formation resulting in quicker bridging and earlier mechanical stability when compared to WT mice, which showed that mature T and B cells are involved in the process (4). Discrepant mechanical characteristics were seen when bone grew with and without B and T cells. The RAG1^−/−^ animals showed stiffer and more brittle bone with less ability to bend and deform when compared to WT bone. This indicates the impact of T and B cells on matrix organization and formation and thus their role in determining bone quality such as the elasticity allowing bone to absorb forces upon impact, thus preventing fractures. The microarray data showed that vitamin D-related gene (Dbp) is upregulated only at D7 and D14 in RAG1^−/−^ compared to WT. Dbp is correlated with T cell proliferation and involved in maintaining the BMD, which supports the decrease in bone quality under the lack of mature T cell.

Faster mineralization in RAG1^−/−^ mice was apparently contradicted by its defective ColI deposition and displacement of Ocn-positive cells in the callus periphery. ColI is the marker ECM molecule of bone, and Ocn is seen as a marker protein for osteoblasts. Therefore, the lack of mature T and B cells has altered two major contributors to bone formation in the RAG1^−/−^ mice. The GBA analysis correlated Spp1, Ctsk, Bglap, Col1a1, and Igf1. Along with the upregulation of Col1a1 at D3 through D7, D14 until D21 those genes suggest the earlier start of mineralization and end of bone formation in the RAG1^−/−^. The osteoclasts, bone cells important for the bone homeostasis and healing as bone degrading cells, showed no differences in numbers up to day 14 of healing. The changes in collagen I deposition found in our analysis are, therefore, not considered connected to an altered osteoclast function. In addition, our analyses of gene expression in bone marrow cells in mice lacking T cells (TCRβδ^−/−^) or B cells (JHT^−/−^) or mature T and B cells (RAG^−/−^) showed no significant difference for OPG and RANKL, both factors involved in osteoclastogenesis.

During endochondral ossification in WT, mineralization starts in the cartilaginous layer with chondrocytes shifting from ColII toward ColX expression. Chondrocytes become hypertrophic and express a number of ECM proteins including osteonectin, fibronectin, and osteopontin (31–33). At this stage, chondrocytes actively express alkaline phosphatase-rich matrix vesicles that are associated with matrix mineralization (34, 35).

Even today tissue mineralization during bone formation is not fully understood (36) but evidence is increasing that mineralization *per se* is not a challenge [as illustrated by ectopic bone formation (37–39), fibrodysplasia ossificans progressiva, or uncontrolled bone formation after limb amputation (40)]. For healthy functional bone to form and to harbor the best quality to withstand strains and stresses of daily life, a controlled, structure-optimizing, and directed bone formation is key to success. Immune cells seem to play an important role in this process (7, 13, 41).

Immune cells are present during the various phases of bone healing (13) (Figure S3 in Supplementary Material). T cells are able to migrate into and through the ECM (42, 43), a process dependent on integrins and actin–myosin activity. Motile cells are thought to be important for the structure of the ECM (44), which is constantly being reorganized in young, transitional tissue, only becoming more stable in mature tissues (45). The major function of the ECM is to provide structural support for cells and tissues, but it is also a storage for growth factors [transforming growth factor β (TGFβ), BMPs, fibroblast growth factor, and insulin-like growth factor] and thus is involved in regulating their bioavailability and in modulating growth signaling events (44). Integrins expressed on T cells can release TGFβ from the ECM *via* their RGD motif (46). With TGFβ being a known regulator of bone formation (47) this indicates the importance of T cells in regulating the bone forming process. Comparing WT and RAG1^−/−^ animal bone healing we saw a faster callus formation from cartilage in the RAG1^−/−^ animals indicating an earlier change toward hypertrophic chondrocytes. TGFβ decreases chondrocyte hypertrophy (48), which could explain the faster maturation in RAG1^−/−^ animals, where TGFβ would be bound in the ECM because the release through T cell action is missing. Another factor which could be responsible for the faster mineralization could be the downregulation of osteopontin (OPN/SPP1) in the RAG1^−/−^ animals. OPN has been reported to inhibit the mineral crystal growth (49) and with a downregulation of OPN being observed in the RAG1^−/−^ animals this could lead to faster callus mineralization. Furthermore, without T cells, TNFα in the fracture callus would be lowered. This pro-inflammatory cytokine has been reported to suppress the expression of bone sialoprotein (BSP) (50). BSP is a crystal nucleator for hydroxyapatite and upon binding of BSP to ColI calcium deposition increases 10-fold (51). Lower TNFα in RAG1^−/−^ animals would cause higher BSP expression and thus increase the calcium deposition on present ColI fibers. Indeed, the data showed lower expression of tumor necrosis factor receptor superfamily, member 11a, NFKB activator, when compared to the WT at D3 and D7 (*p* = 0.0102 and *p* = 0.0071, respectively). The above are some explanations on how the regulation of T cells impacts matrix mineralization and concurs with our results in animals lacking mature T and B cells. T cells appear to affect cartilage mineralization by slowing the process itself down (when compared to lymphopenic mice), giving matrix organization and mineral deposition adequate time to enable callus structure to concomitant mechanical strains.

In the RAG1^−/−^ animals, we detected lower Ocn expression in the fracture callus. With Ocn binding to hydroxyapatite this could indicate a disturbed bone healing, however, Ocn knockout animals only show a mild bone phenotype and prove that mineralization without Ocn is still possible (52). Ocn, however, is also a marker for osteoblasts and the altered distribution of these cells in the RAG1^−/−^ animal fracture callus indicates another role for T cells in the bone healing process. T cells seem to be essential for osteoblast maturation. Expression of IL17F and wingless-type MMTV integration site family, member 10B by T cells has been reported to be essential in the osteoblasts maturation process (10, 53). The close spatial proximity observed between T and B cells and osteoprogenitors in the bone marrow previously reported from our group (13) is another confirmation for the interdependency of these cells. It cannot be excluded that T cell-specific surface molecules like CTLA-4 (12) and its ligands CD80 and CD86 on the surface of osteoblasts (54) might be involved. It could be argued that our result depends on an indirect effect on B cells due to the lack of CD40-CD40L engagement between these and T cells. This pathway has been shown to be important for bone homeostasis (55). However, if this was the case, the phenotype of T cell- and B cell-deficient mice should have overlapped. Still, it has been shown that murine primary osteoblasts do express CD40 and that its engagement protects them from TNFα-induced apoptosis (56). It seems conceivable that lack of CD40-CD40L engagement between T cells and osteoblast precursors would have a major effect on ColI deposition by these cells in the soft callus.

The curious finding that Ocn-positive cells line up on the outside of the RAG1^−/−^ fracture callus could be an indication of another function of immune cells during the bone healing process. Previous studies showed that the lymphatic draining in the injured bone is activated upon fracture and that prolonged lymphonodal activity is associated with successful healing (57, 58). Ocn -positive cells accumulate in the peripheral blood after an injury (59) and osteoblastic precursors have been reported to invade fractured bone *via* blood vessels (60). However, it is unclear whether osteoblast precursors, which reach the callus from vasculature of the surrounding soft tissues (Figure 2), require interaction with lymphocytes in secondary lymphoid organs or *in situ* for proper trafficking. Further studies should be carried out to elucidate the complete mobilization pathway of these cells during bone repair. This is in accordance with the upregulation of VEGF and the downregulation of Serpinf1 as seen at D7 and D14, which both play a role in osteoblast regulation (61).

To partake in the healing process, osteoblastic cells would be required to invade the fracture side which directly after the injury would be filled with a hematoma. The hematoma has an unfavorable milieu for cells to survive and being active due to the low oxygen levels, lower pH value, and higher Na^+^ and K^+^ values (62–64), however, immune cells, including T cells, are able to survive and thrive in this surrounding (65). Therefore, the immune cells are essential for the chemoattractant pattern responsible for cell recruitment during this early stage of healing (66). Among those cells recruited during early healing phases are again immune cells, including T cells. With the above finding, it can be hypothesized that T cells are essential players in orchestrating the matrix forming osteoblasts, specifically in the phase of endochondral ossification. With T cells missing at this early stage the cellular composition during initiation of healing is altered, the ECM that is formed and necessary for cell invasion is, therefore, different and perhaps is no longer favorable for invasion of Ocn-positive cells. This would indicate that an important source of osteoblast precursors cannot be accessed in mature T- and B cell deficient mice. The Ocn-postive cells stay lined up outside of the fracture callus.

## Conclusion

This work for the first time presents a direct link between immune cells and matrix formation during regeneration using bone healing as an example. It illustrates specifically the role of T cells in the collagen organization process and the lack thereof in the absence of T cells.

The importance of immune cells during the fracture healing process has already been established. Here, we show that immune cells play an essential part in the regulation of the mineralization in endochondral ossification, which is necessary to generate bone with an elastic quality able to withstand forces. In detail, immune cells are needed to slow down the mineralization process in order to build quality bone, and immune cells are needed to recruit osteogenic precursors and aid their differentiation.

Even though callus formation in itself is faster without T and B cells the ensuing bone callus and organ tissue do not have the sufficient quality and thus functionality that is achieved when these immune cells take their active role in the bone organization and formation process.

## Ethics Statement

This study was carried out in accordance with the recommendations of the Animal Welfare Act, the National Institutes of Health Guide for Care and Use of Laboratory Animals, and the National Animal Welfare Guidelines and was approved by the local legal representative animal rights protection authorities: Landesamt für Gesundheit und Soziales Berlin. The protocol was approved by the Landesamt für Gesundheit und Soziales Berlin: G 0206/08.

## Author Contributions

TK contributed data acquisition: data evaluation, study design, and manuscript writing; AS contributed data acquisition: histology, data evaluation, and manuscript writing; AP contributed data acquisition: second harmonic imaging and manuscript writing; CB contributed data acquisition: biomechanics, cell culture, and data evaluation; CS contributed data acquisition: histology and manuscript writing; IK contributed data acquisition: histology, data evaluation, and study design; DM contributed data acquisition: data evaluation; SW contributed data acquisition: in cell culture and ELISA, data evaluation, and manuscript writing; HS contributed study design, manuscript writing; H-DV contributed to study design and manuscript writing; KS-B contributed data acquisition: biomechanics study design, data evaluation, and manuscript writing; GD contributed study design and manuscript writing.

## Conflict of Interest Statement

The authors declare that the research was conducted in the absence of any commercial or financial relationships that could be construed as a potential conflict of interest.
