# Supplementary material for: T Lymphocytes Influence the Mineralization Process of Bone
Source: Front Immunol. 2017 May 24;8:562. doi: 10.3389/fimmu.2017.00562 (PMC5442173; doi:10.3389/fimmu.2017.00562)
Supplement: Supplementary file 1 [file Presentation_1.pdf]

Supplemantary figures and tables:

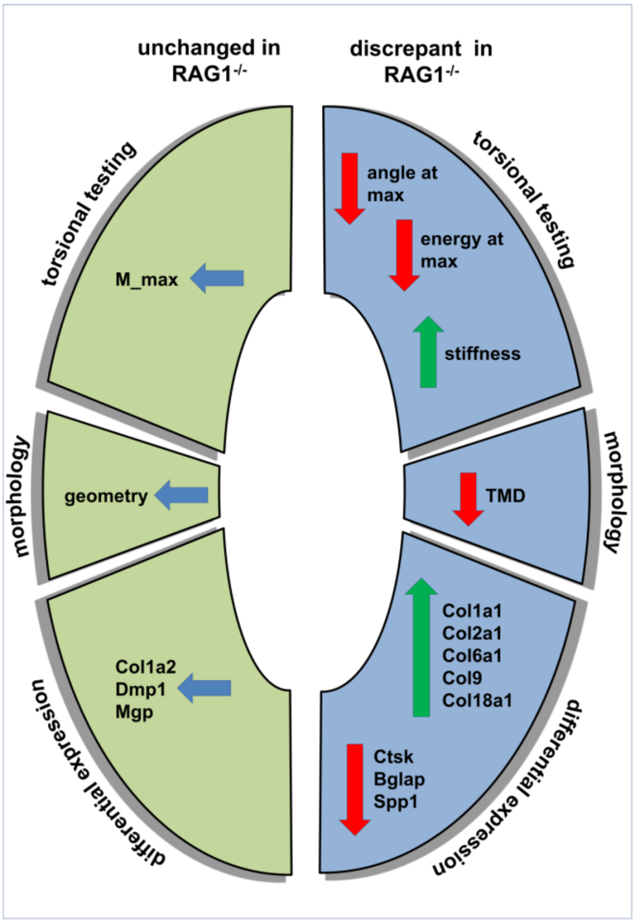

Figure S1: Overall structural and functional competence in intact RAG1$^{-/-}$ bone is affected by differential expression but not morphology. Histological and microCT analyses showed no differences in femur morphology and geometry between both mice types. Structural analysis showed a decrase of TMD in the RAG1$^{-/-}$ mice (p=0.001). ECM related genes such as collagen components were up-regulated in the RAG1$^{-/-}$ mice bone, whereas other matrix proteins saw no expression changes. Moreover, cellular markers especially those related to resorption such as cathepsin K were down-regulated in the RAG1$^{-/-}$. Altogether, these intrinsic factors reflect the main function of bone showing higher stiffness that requires smaller angle and less energy up to maximum torque at failure. Biomechanical analysis revealed no differences in maximum torque at failure between RAG1$^{-/-}$ and WT. Detailed data and network analysis of the initial discrepancies between the groups are shown in the figures 1 and 2.

Table S1: Differentially expressed genes in intact WT and RAG1$^{-/-}$ bones

time point		gene	full name	fold change	P-value
D0	Up regulated	Col1a1	Collagen type 1 alpha 1	+2.05	0.0064
		Col2a1	Collagen type 2 alpha 1	+1.448	0.0047
		Col6a1	Collagen type 6 alpha 1	+1.013	0.003
		Col9a2	Collagen type 9 alpha 2	+1.876	0.005
		Col18a1	Collagen type 18 alpha 1	+1.400	0.010
	Down regulated	Ctsk	Cathepsin K	-1.474	0.009
		Bglap	Bone Gamma Carboxyglutamate Protein	-1.889	0.001
		Spp1	Secreted Phosphoprotein 1	-1.444	0.04
D3	Up regulated	Col1a1	Collagen type 1 alpha 1	+2.318	0.0031
		Col2a1	Collagen type 2 alpha 1	+1.033	0.011
		Col6a1	Collagen type 6 alpha 1	+2.614	0.0006
		Col18a1	Collagen type 18 alpha 1	+1.107	0.0022
		Mgp	Matrix Gla protein	+1.943	0.0016
	Down regulated	Ctsk	Cathepsin K	-1.644	0.0013
		Spp1	Secreted Phosphoprotein 1	-1.528	0.003
D7	Up regulated	Col1a1	Collagen type 1 alpha 1	+1.612	0.0062
		Col2a1	Collagen type 2 alpha 1	+1.062	0.004
		Col6a1	Collagen type 6 alpha 1	+2.388	0.0004
		Col9a2	Collagen type 9 alpha 2	+2.213	0.0006
		Col18a1	Collagen type 18 alpha 1	+1.607	0.021
		Mgp	Matrix Gla protein	+1.837	0.0008
	Down regulated	Col1a2	Collagen type 1 alpha 2	-1.888	0.0064
D14	Down regulated	Col2a1	Collagen type 2 alpha 1	-1.824	0.0037
		Col9a2	Collagen type 9 alpha 2	-1.155	0.017
		Bglap	Bone Gamma Carboxyglutamate Protein	-1.684	0.0012
		Mgp	Matrix Gla protein	-2.126	0.0012
D21	Up regulated	Col1a1	Collagen type 1 alpha 1	+1.13	0.0027
		Col2a1	Collagen type 2 alpha 1	+2.386	0.002
		Col6a1	Collagen type 6 alpha 1	+1.034	0.007
		Col9a2	Collagen type 9 alpha 2	+2.585	0.005
D28	Up regulated	Col1a2	Collagen type 1 alpha 2	+1.183	0.030
	Down regulated	Ctsk	Cathepsin K	-1.493	0.003

Table S2: Expression data and p values completing Figure 5 G and H

time point		gene	full name	fold change	P-value
D0	Up regulated	Col1a1	Collagen type 1 alpha 1	+2.05	0.0064
		Col1a2	Collagen type 1 alpha 2	+0.99	0.06
D3	Up regulated	Col1a1	Collagen type 1 alpha 1	+2.318	0.0031
	Down regulated	Col1a2	Collagen type 1 alpha 2	-0.976	0.012
D7	Up Regulated	Col1a1	Collagen type 1 alpha 1	+1.612	0.0062
	Down regulated	Col1a2	Collagen type 1 alpha 2	-1.888	0.0064
D14	Down regulated	Col1a2	Collagen type 1 alpha 2	-0.472	0.03
D21	Up regulated	Col1a1	Collagen type 1 alpha 1	+1.13	0.0027
		Col1a2	Collagen type 1 alpha 2	+0.849	0.04
D28	Up regulated	Col1a2	Collagen type 1 alpha 2	+1.183	0.030

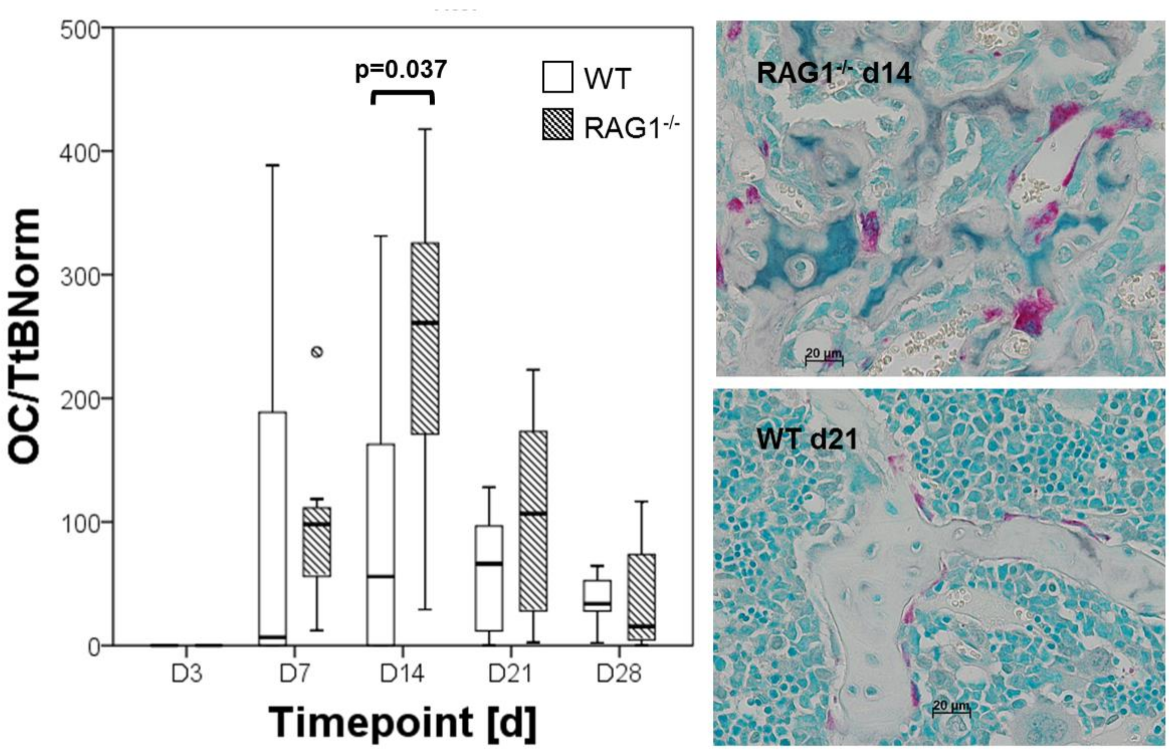

Figure S2: Osteoclasts numbers in bone healing with and without mature T and B cells: No differences in osteoclasts numbers were found except for day 14 where osteoclasts were significantly more abundant in $RAG^{-/-}$ animals. On the right side example images of osteoclasts are depicted for $RAG^{-/-}$ at day 14 and WT at day 21 of healing.

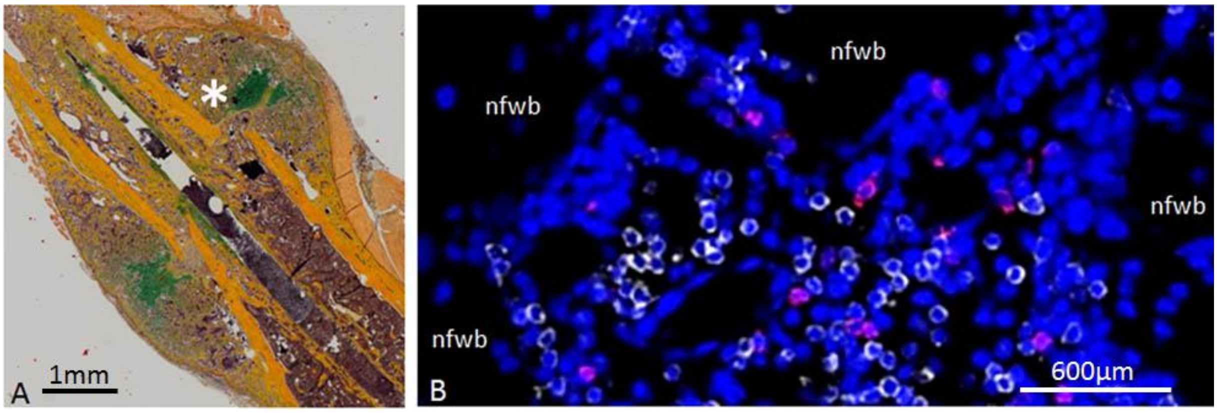

Figure S3: T and B cells are present in the fracture callus of WT animals: WT fracture callus 14 days after fracture, immune histology shows the area marked with *. B cells are white (B220), T cells are red (CD3), and nuclei are stained blue with DAPI, nfwb marks newly formed woven bone.

Table S3 Fracture callus mineralization – area values

		periosteal	endosteal
		µm^2	µm^2
7 days	WT red	11	11
	Rag red	51	17
14 days	WT red	96	182
	Rag red	108	63
	WT green	40	92
	Rag green	31	34
21 days	WT red	4	16
	Rag red	37	20
	WT green	13	36
	Rag green	1	30
	WT blue	40	39
	Rag blue	6	6
